# A defined isothiocyanate mixture suppresses platelet signaling and metabolism to limit thrombosis *in vivo*

**DOI:** 10.3389/fimmu.2026.1944710

**Published:** 2026-09-30

**Authors:** Jan Yağız Buchholz, Maria Shoykhet, Anthea C. Darius, Tobias Weiser, Erika Lynn Roberts, Mohammad Khosravi, Julie Petry, Seyedeh Zahra Mousavi, Babak Mohammadian, Lina Adams, Khouloud Hachani, Carola Eberhagen, Omid Azimzadeh, Hans Zischka, Gabriele Multhoff, Ali Bashiri Dezfouli, Barbara Wollenberg

**Affiliations:** 1 Department of Otorhinolaryngology, TUM School of Medicine and Health, Technical University of Munich (TUM), Munich, Germany; 2 Central Institute for Translational Cancer Research, Technical University of Munich (TranslaTUM), Klinikum rechts der Isar, Munich, Germany; 3 Department of Pathobiology, Faculty of Veterinary Medicine, Shahid Chamran University of Ahvaz, Ahvaz, Iran; 4 Institute of Molecular Toxicology and Pharmacology, Helmholtz Center Munich, German Research Center for Environmental Health, Neuherberg, Germany; 5 Section Radiation Biology, Federal Office for Radiation Protection (BfS), Neuherberg, Germany; 6 Institute of Toxicology and Environmental Hygiene, Technical University of Munich School of Medicine and Health, Munich, Germany

**Keywords:** antithrombotic therapy, isothiocyanates (ITCs), mitochondrial dysfunction, oxidative stress, platelet activation, platelet aggregation

## Abstract

Pathological platelet activation drives thrombosis and is a major contributor to morbidity and mortality. Current antiplatelet therapies reduce thrombotic risk but target a limited range of signaling pathways, have minimal impact on established platelet aggregates, and are frequently associated with bleeding complications. These drawbacks have prompted interest in alternative antiplatelet strategies. Isothiocyanates (ITCs), phytochemicals from cruciferous plants, have been reported to inhibit platelet function; however, their mechanisms and translational relevance remain incompletely defined. The effects of a defined mixture of allyl-, benzyl-, and phenethyl-isothiocyanate on platelet function were evaluated using *ex vivo* human platelets and an *in vivo* murine model of carrageenan-induced thrombosis. Platelet aggregation, intracellular signaling, calcium mobilization, redox balance, and mitochondrial function were assessed using functional assays, kinome profiling, flow cytometry, respirometry, and electron microscopy. The ITC mixture was administered before or after the carrageenan challenge. The ITC mixture inhibited platelet activation and aggregation induced by multiple agonists. It reduced peptide phosphorylation across multiple platelet signaling pathways, reduced intracellular calcium mobilization and the activation-associated oxidant signal, and impaired mitochondrial function. When the ITC mixture was added shortly after activation, aggregometry signals consistent with destabilization of early platelet aggregates were observed whereas granule secretion was irreversible once initiated. *In vivo*, ITC mixture administration before or after the carrageenan challenge reduced thrombus formation and tissue injury, prolonged bleeding times, and reduced tail-necrosis length to a similar extent as the heparin reference group in this model. These findings identify the ITC mixture as a modulator of platelet signaling and metabolism, provide mechanistic insight into ITC-mediated antithrombotic effects, and support further evaluation of ITC-based interventions in thrombotic disease.

## Graphical abstract

**Figure:**
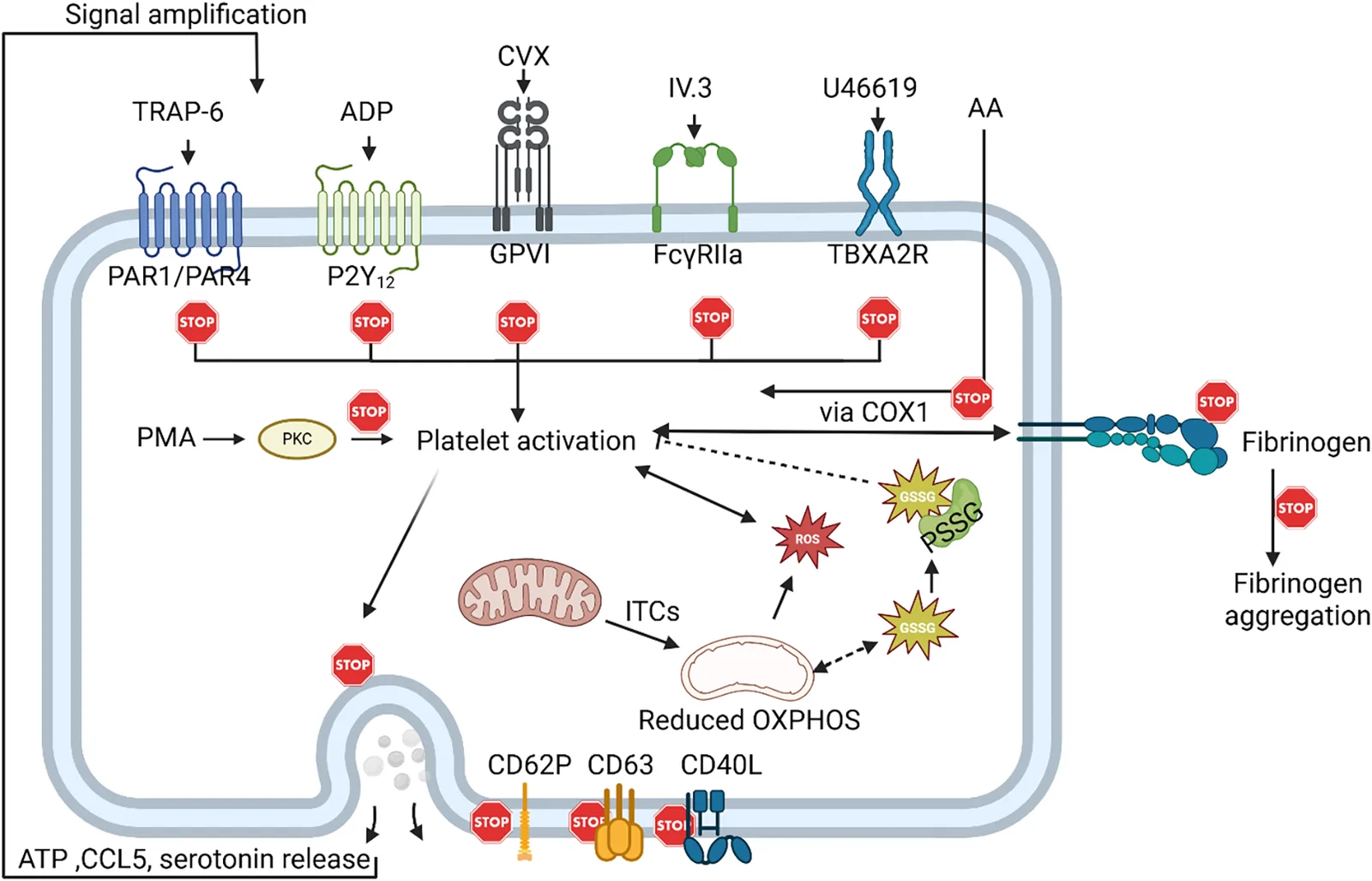
The graphical abstract illustrates the multimodal antiplatelet mechanism of the ITC mixture. Platelet activation is initiated by diverse agonists acting through G-protein-coupled and immunoreceptor pathways, including ADP (P2Y_12_) the PAR1/PAR4-activating peptide TRAP-6, U46619 (thromboxane A_2_ receptor), convulxin (GPVI), IV.3-mediated FcγRIIa crosslinking, arachidonic acid (COX-1-dependent) and PMA (direct PKC activation), which collectively converge to amplify platelet signaling. Serotonin is depicted as a dense-granule release product quantified in this study, and not as an agonist applied to platelets; thrombin receptor signaling was addressed using TRAP-6 rather than thrombin itself. Centrally, the ITC mixture impairs mitochondrial oxidative phosphorylation and disrupts redox homeostasis, leading to altered redox homeostasis and activation-associated oxidant signaling, thereby limiting the energetic support required for sustained platelet activation. Consequently, granule secretion and surface expression of activation markers (CD62P, CD63, CD40L) are reduced, resulting in diminished integrin activation, impaired fibrinogen binding, and attenuated platelet aggregation. *Created in BioRender. Hachani, K (2026).* *https://BioRender.com/9exdvj3*.

## 1 Introduction

Platelets are anucleate cell fragments derived from bone marrow megakaryocytes and are central regulators of vascular hemostasis and thrombosis (1). Under physiological conditions, platelets remain in a quiescent, non-adhesive state maintained by endothelial-derived mediators such as prostacyclin (PGI_2_), nitric oxide (NO), and a tightly regulated mitochondrial energy program (2–4). Following vascular injury, platelets are rapidly activated by extracellular agonists, including collagen and adenosine diphosphate (ADP), and, in immune-mediated contexts, by immune complexes engaging platelet surface receptors. These stimuli trigger intracellular signaling cascades involving phosphoinositide 3-kinase/protein kinase B (PI3K/AKT), protein kinase C (PKC), and related kinase pathways, which coordinate cytoskeletal remodeling, integrin activation, granule secretion, and firm adhesion (5–8). Upon activation, platelets release cytokines and pro-thrombotic mediators, establishing positive feedback loops that further amplify platelet recruitment and activation (9, 10). Although this process is essential for physiological hemostasis, dysregulated platelet activation contributes to pathological thrombosis, a major cause of myocardial infarction, stroke, pulmonary embolism, and multi-organ failure (11–14).

Despite the widespread clinical use of antithrombotic drugs to combat such complications, major limitations persist (15–17). Most clinically used agents target a single receptor or enzymatic pathway, such as cyclooxygenase inhibition by aspirin or P2Y_12_ blockade by ADP receptor antagonists, leaving alternative activation routes intact. As a result, residual platelet reactivity and thrombotic risk remain in many patients. Moreover, most of these agents suppress platelet activation or aggregation but do not actively dissolve pre-existing platelet aggregates, largely restricting true thrombolysis to plasminogen activators (17–20). In addition, inter-individual variability in platelet reactivity and incomplete platelet inhibition contribute to residual thrombotic risk even in treated patients (15). These shortcomings support the need for novel antithrombotic strategies that more broadly suppress platelet activation and, ideally, act on aggregates that have already begun to form, while maintaining an acceptable safety profile.

Platelet activation is an energetically demanding and tightly regulated process that integrates intracellular signaling, calcium mobilization, redox balance, and mitochondrial metabolism to culminate in the formation of a stable hemostatic plug (14, 21). Reactive oxygen species (ROS) play a critical role as secondary messengers in this activation, particularly downstream of collagen stimulation, where oxidative modification of signaling proteins amplifies platelet responses (22, 23). Mitochondria integrate these energetic and redox processes by supplying the ATP required for granule secretion and cytoskeletal remodeling, while simultaneously regulating ROS generation through the electron transport chain (21). However, excessive oxidative stress may paradoxically impair platelet function and facilitate receptor shedding via mechanisms dependent on p38 mitogen-activated protein kinase (p38 MAPK) (23). These observations highlight platelet metabolism and redox regulation as attractive but underexplored targets for antithrombotic intervention.

Due to their pleiotropic biological activities and generally favorable safety profiles, naturally occurring phytochemicals have emerged as promising candidates for antithrombotic intervention. Among these, isothiocyanates (ITCs), bioactive compounds derived from glucosinolates found in cruciferous plants such as mustard seed and horseradish root (allyl-ITC), garden cress and papaya seed (benzyl-ITC), watercress (phenethyl-ITC), and broccoli and broccoli sprouts (sulforaphane), in which the parent glucosinolates are hydrolyzed to the corresponding ITCs by myrosinase upon tissue damage during chopping, chewing or food processing, have long been used in phytomedicine for their antimicrobial, anti-inflammatory, and cytoprotective properties (24, 25). Clinically, ITCs are utilized in a herbal formulation known as ANGOCIN^®^ *Anti-Infekt N* (Angocin) (26), which contains benzyl isothiocyanate (BITC), allyl isothiocyanate (AITC), and phenethyl isothiocyanate (PEITC), and is prescribed for respiratory and urinary tract infections (27, 28). Beyond their antimicrobial activity, ITCs exert potent anti-inflammatory effects through inhibition of NF-κB and NLRP3 inflammasome signaling in immune cells (29–32).

ITCs are also well recognized for their ability to modulate mitochondrial function and cellular redox balance. In cancer cells, PEITC and BITC induce apoptosis via ROS-dependent mitochondrial membrane depolarization, cytochrome c release, and caspase activation (33–36). In contrast, ITCs exert antioxidant and cytoprotective effects in endothelial cells and astrocytes by enhancing Nrf2-dependent defense systems and suppressing inflammatory signaling (37, 38). These context-dependent redox effects, together with the capacity of ITCs to regulate inflammatory signaling and mitochondrial metabolism, intersect with several core regulators of platelet activation, including ROS signaling, calcium mobilization, PI3K/AKT signaling, and mitochondrial bioenergetics.

Consistent with this conceptual overlap, several ITCs have been reported to inhibit platelet aggregation. Sulforaphane (SFN) suppresses platelet activation and coagulation factor activity and protects against cerebral thrombotic dysfunction *in vivo* (39, 40), while AITC and isopropyl ITC inhibit platelet aggregation and thrombus formation in experimental models (41–43). Nevertheless, the molecular mechanisms underlying ITC-mediated platelet inhibition remain incompletely understood, especially regarding effects on platelet redox balance, calcium signaling, and mitochondrial function. Moreover, although ITCs are commonly administered clinically as defined combinations, no previous study has comprehensively examined whether ITCs exert synergistic or mechanistically distinct effects on platelet activation and thrombosis.

In the present study, we investigated the effects of a defined mixture of AITC, BITC, and PEITC on platelet activation and aggregation in *ex vivo* human platelets and in an *in vivo* murine thrombosis model. We show that the ITC mixture inhibits platelet aggregation, partially suppresses platelet activation and degranulation, and destabilizes early, already-forming platelet aggregates. Mechanistically, ITC treatment attenuates key activation pathways, including AKT phosphorylation and upstream kinase signaling, reduces intracellular Ca^2+^ mobilization, and reduces the activation-associated oxidant signal. In parallel, the ITC mixture is associated with oxidative stress and mitochondrial dysfunction in platelets, as reflected by glutathione oxidation, increased protein S-glutathionylation, loss of mitochondrial membrane potential, and impaired mitochondrial respiration. *In vivo*, administration of the ITC mixture before or after the carrageenan challenge reduced thrombus formation and tissue injury, with reductions in the measured thrombosis endpoint of a similar magnitude to those observed in the heparin reference group, and a similar prolongation of bleeding time.

These findings identify this ITC mixture as a dual regulator of platelet signaling and metabolic support systems and reveal a previously unrecognized mechanism by which this ITC mixture suppresses platelet-driven thrombosis. This work establishes a mechanistic foundation for the development of ITC-based antithrombotic strategies and provides a framework for future translational studies aimed at evaluating their therapeutic potential in thrombotic disease.

## 2 Materials and methods

### 2.1 Platelet preparation and treatments

Human platelet studies were approved by the Ethics Committee of the Technical University of Munich (approval numbers 2020-474_2-S-NP and 2022-211-S-NP). All blood donors provided written informed consent. Platelets were obtained from unrelated healthy volunteers of mixed gender, aged 25–35 years. All donors were of self-reported good health with no acute infection or inflammatory condition at the time of donation, and had not taken any medication known to affect platelet function (including non-steroidal anti-inflammatory drugs) for at least 10 days before blood sampling. All donors were non-smokers and none reported regular alcohol consumption. Ethnicity and ancestry were not systematically recorded. All donors were recruited in Munich, Germany. The number of independent donors contributing to each analysis is reported in **Supplementary Table S1**; these panel-level values are numbers of independent biological replicates and do not represent the total number of unique donors across the study.

#### 2.1.1 Platelet isolation

Platelet isolation was performed as previously described in established platelet isolation protocols (44, 45). Peripheral blood from healthy donors was collected in 3.2% sodium citrate tubes and processed within 2 hours of venipuncture. Platelet-rich plasma (PRP) was generated by centrifugation at 150 × g for 20 minutes at room temperature (RT, ~22 °C). To prevent inadvertent activation, PRP was supplemented with prostacyclin (PGI_2_, 400 nM) and centrifuged at 800 × g for 10 min. The resulting platelet pellet was gently resuspended in Ca^2+^-free Tyrode buffer (pH 7.4) (46). Platelet counts and purity (CD41^+^/CD45^-^) were assessed by flow cytometry.

#### 2.1.2 ITC mixture preparation

The ITC mixture consisted of 37.9% allyl-ITC (AITC; #377430, Sigma-Aldrich), 50.0% benzyl-ITC (BITC; #252492, Sigma-Aldrich), and 12.1% phenethyl-ITC (PEITC; #253731, Sigma-Aldrich). The relative proportions of the three compounds were fixed *a priori* to reflect the relative isothiocyanate content of the clinical preparation ANGOCIN^®^ Anti-Infekt N (26, 47), and were not optimized for antiplatelet activity in the present study. A concentrated stock solution of the ITC mixture was prepared in dimethyl sulfoxide (DMSO) and subsequently diluted into assay buffers for experimental use. Control samples received matched DMSO concentrations for *in vitro* experiments (final < 0.2% v/v). In every experiment reported, the control condition was the corresponding dilution buffer carrying the matched DMSO concentration. Inhibitory effects of DMSO on human platelet adhesion and aggregation have been described at concentrations of 0.5% v/v and above (48), i.e. more than twofold above the maximum concentration reached in the present study. For *in vitro* experiments, the mixture was applied at a total concentration of 845 µM (320 µM AITC, 422.5 µM BITC, 102.5 µM PEITC), a dose selected from dose-response experiments demonstrating complete inhibition without cytotoxicity.

#### 2.1.3 Platelet agonists and ITC timing protocols

Platelets were stimulated with the following agonists at their final concentrations: TRAP-6 (25 µM; #3497, Tocris), convulxin (CVX; 125 ng/mL; #119-02, Pentapharm), U46619 (1 µM; #16450, Cayman), arachidonic acid (AA; 100 µM; #23401, Sigma-Aldrich), ADP (100 µM; #A2754, Sigma-Aldrich), and PMA (1 µM; #P1585, Sigma-Aldrich). For FcγRIIa-mediated activation, platelets were incubated with the monoclonal antibody IV.3 (383 ng/mL), generously provided by Ronald Taylor (University of Virginia), and cross-linked using anti-Fc Fab (15 µg/mL; #115-006-062, Jackson ImmunoResearch).

To assess the timing-dependent effects of the ITC mixture, two experimental paradigms were employed. For pre-treatment experiments, platelets were incubated with the ITCs for 5 minutes prior to agonist stimulation. For post-activation (reversal) protocols, platelets were first activated with the indicated agonist, after which the ITC mixture was added 25 or 300 s post-stimulation, as specified in the corresponding figure legends. These post-activation addition experiments were designed to evaluate whether the ITC mixture can modulate ongoing platelet activation under experimental conditions.

### 2.2 Functional platelet assays

#### 2.2.1 Light transmission aggregometry and cytotoxicity

Platelet aggregation was assessed by light transmission aggregometry using the Born method on an APACT 4S Plus aggregometer (DiaSys) (49). Platelets were adjusted to 2 × 10^8^ platelets/mL and stirred at 1000 rpm at 37 °C. Aggregation was initiated by agonist addition and monitored immediately for up to 10 min, after which maximal aggregation and area under the curve (AUC) were quantified. Cytotoxicity was evaluated using Annexin V-APC staining to detect phosphatidylserine exposure after 5 minutes of exposure to the ITC mixture, i.e. the pre-treatment interval used in the aggregation experiments. Plasma-membrane integrity was assessed in parallel by lactate dehydrogenase (LDH) release, measured across the full range of exposure intervals used in this study (300, 900 and 1800 s). The assay is described in the Supplementary and the results are shown in **Supplementary Figure S1**, **Supporting Information**.

#### 2.2.2 Flow cytometry

Flow cytometric analysis was performed on a CytoFLEX (Beckman Coulter). At least 20,000 events were collected per sample. Platelets were identified by FSC/SSC profile and CD41^+^/CD45^-^ gating. Activation markers included CD62P-BV510 (P-selectin; #304936, Biolegend), CD63-BV421 (#353029, Biolegend), and CD154-PE/Cy7 (CD40L; #310831, Biolegend) as previously described (50). Integrin activation was assessed using PAC-1-FITC (#362804, Biolegend). Fibrinogen binding was measured using Alexa Fluor 488-conjugated fibrinogen (#F13191, Thermo Fisher). Apoptosis was detected with Annexin V-APC (#640932, Biolegend). Compensation beads (BD CompBeads, #552843) were used to prepare single-stain compensation controls.

#### 2.2.3 Release assays

α-granule release was quantified by CCL5 ELISA (#440804, Biolegend). Dense-granule release was measured by ATP quantification (#G924, Promega) and serotonin ELISA (#DEE8900, Demeditec). Supernatants were collected immediately after stimulation, centrifuged, and analyzed using standard curves. Values were normalized based on the supernatant volume derived from equivalent platelet counts.

### 2.3 Mechanistic intracellular assays

#### 2.3.1 Intracellular ROS and glutathione

ROS levels were measured using CellROX™ Green (5 µM; #C10444, Thermo Fisher) and reported as geometric mean fluorescence intensity (gMFI), normalized to unstimulated controls. Total and oxidized glutathione (GSH, GSSG) were quantified using the GSH/GSSG-Glo assay (#V6611, Promega) and expressed relative to unstimulated controls. Protein S-glutathionylation (PSSG) was measured using a Cayman probe kit (#Cay10010721-1) and reported as gMFI. All assays were performed according to manufacturer’s protocols. Because retention of CellROX™ Green is influenced by the mitochondrial membrane potential, the ROS measurements were repeated using CellROX™ Orange (#C10443, Thermo Fisher), a cell-permeant probe that is retained in the cytosol upon oxidation and does not accumulate in the nucleus or in mitochondria. This assay is described in the Supplementary and the results are shown in **Supplementary Figure S2**, **Supporting Information**.

#### 2.3.2 Calcium measurements

Platelets were loaded with Calbryte-520 AM (5 µM; #ABD-20650, AAT Bioquest) for 1 hour at 37 °C in the dark, washed, and analyzed using a fluorescence reader (Ex 490–500 nm/Em 515–525 nm) similar to previously established platelet protocols (46). Fluorescence was baseline-corrected to the 10 s pre-stimulation interval, and measurements were acquired every 7.5 s for 5 minutes.

#### 2.3.3 Mitochondrial membrane potential (Δψm)

Δψm was assessed using Tetramethylrhodamin-methylester (TMRM, 200 nM; #87917, Sigma-Aldrich) in non-quenching mode as previously optimized for platelet protocols (52). Platelets were incubated with TMRM for 20 minutes at 37 °C. Carbonyl cyanide m-chlorophenyl hydrazone (CCCP, 10 µM; #C2759, Sigma-Aldrich) was used as a positive control for depolarization.

#### 2.3.4 High-resolution respirometry

Mitochondrial respiration was measured using high-resolution respirometry on an Oxygraph-2k (Oroboros Instruments) following the substrate-uncoupler-inhibitor titration (SUIT) protocol optimized for platelets (52). Platelets (3 × 10^8^) were suspended in 2 ml MiR05 medium. The following respiratory states were quantified: routine respiration of intact platelets; leak respiration after plasma-membrane permeabilization with digitonin (1 µg per 10 µg platelet protein; #HN76.2, Carl Roth); CI-linked leak respiration after subsequent addition of malate (2 mM) and pyruvate (5 mM); CI-linked OXPHOS capacity after addition of ADP (2.5 mM); CI-linked electron transfer system (ETS) capacity after uncoupling with CCCP (0.5 µM; #C2759, Sigma-Aldrich); NADH-linked ETS capacity after addition of glutamate (10 mM); convergent CI- and CII-linked ETS capacity after addition of succinate (10 mM; #sc-251053, SCBT); CII-linked ETS capacity after inhibition of complex I with rotenone (0.5 µM; #T2970, TargetMol); GPDH-linked ETS capacity after addition of glycerol-3-phosphate (10 mM); and residual oxygen consumption (ROX) after inhibition of complex III with antimycin A (2.5 µM; #A8674, Sigma-Aldrich). Oxygen fluxes were normalized to protein content measured by Bradford assay.

#### 2.3.5 Transmission electron microscopy

Platelets were fixed in 2.5% glutaraldehyde in 0.1 M sodium cacodylate buffer, post-fixed in osmium tetroxide, dehydrated in acetone, and embedded in epoxy resin. Ultrathin sections (50–60 nm) were stained with UranyLess and 3% lead citrate and imaged using a Jeol 1200 EXII transmission electron microscope and processed with the iTEM software package (analySISFive, Olympus). For each condition, at least 50 mitochondria from ≥30 platelets were evaluated in a blinded manner. Mitochondrial area and cristae counts were quantified using the QuPath software.

### 2.4 Signaling analysis

#### 2.4.1 Kinome profiling (PamGene)

Kinase activity was assessed using the PamGene PamChip^®^ platform, comprising both the serine/threonine kinase (STK) and the protein tyrosine kinase (PTK) arrays. Platelet lysates from control (300 s), TRAP-stimulated platelets (300 s TRAP), and platelets treated with ITCs either prior to TRAP stimulation (300 s ITC pre-treatment followed by 300 s TRAP stimulation) or following TRAP activation (325 s TRAP stimulation, with the ITC mixture added 25 s after TRAP) groups were applied to PamChip^®^ microarrays Phosphorylation signals were analyzed using BioNavigator software. Kinase predictions were considered significant at p < 0.05 with Benjamini-Hochberg false discovery rate correction.

#### 2.4.2 Kinome data visualization and bioinformatic analysis

Differential phosphorylation results from PamGene kinome profiling were visualized using volcano plots generated with the Enhanced Volcano package (v1.20.0) in R. Volcano plots display log_2_ fold change versus −log_10_ adjusted p value for the indicated pairwise comparisons, including ITC alone treatment versus control, TRAP alone treatment versus ITC pre-treatment followed by TRAP stimulation (ITC+TRAP), and TRAP stimulation followed by ITC post-treatment (TRAP+ITC) conditions.

For global visualization of phosphorylation patterns, log_2_-transformed phosphorylation intensities for 242 phosphosites across 30 samples (six biological replicates per group across five experimental conditions: control, TRAP, ITC, ITC+TRAP, and TRAP+ITC) were visualized as clustered heatmaps using the pheatmap package (v1.0.12) in R. Values were column-wise z-score normalized (mean-centered and scaled to standard deviation for each phosphosite). Hierarchical clustering was applied to phosphosites using Euclidean distance and complete linkage.

#### 2.4.3 Western blotting

Platelets were pretreated with the ITC mixture or vehicle control for 300 s, followed by stimulation with TRAP. In reversal experiments, platelets were initially stimulated with TRAP, and the ITC mixture was added 25 s or 300 s after stimulation. The total incubation time for all experimental conditions was 900 s. Platelets were then lysed in ice-cold lysis buffer (#9803, Cell Signaling Technology) supplemented with Protease/Phosphatase Inhibitor Cocktail (#5872, Cell Signaling Technology). Equal protein amounts (30 µg) were separated by SDS-PAGE, transferred to PVDF membranes, and probed for phospho-AKT (Ser473; #4060, Cell Signaling Technology), total AKT (#9272, Cell Signaling Technology), GAPDH (#607902, BioLegend), α-tubulin (MA1-80189, Thermo Fisher Scientific). Detection was performed using fluorophore-conjugated secondary antibodies: donkey anti-rat IgG (H+L) AlexaFluor 555 (#A78945, Thermo Fisher Scientific), and anti-rabbit IgG (H+L) DyLight 680 conjugate (#5366, Cell Signaling Technology). All protein signals were detected and quantified using the LI-COR Odyssey Fc imaging system.

### 2.5 *In vivo* thrombosis model and animal experiments

#### 2.5.1 Animal handling and ethical approval

Healthy male BALB/c mice (7–9 weeks of age, 25 ± 5 g) were purchased from the Pasteur Institute (Tehran, Iran). Animals were housed under controlled environmental conditions (12 hours light/dark cycle, 22 °C, 55% relative humidity) with ad libitum access to standard laboratory chow and water. Mice were allowed to acclimatize for a minimum of two weeks before the start of experiments. All animal procedures were conducted in accordance with the revised European Union Directive 2010/63/EU for the protection of animals used for scientific purposes and the Guidelines for the Care and Use of Laboratory Animals. Experimental protocols were approved by the Animal Ethics Committee of Shahid Chamran University of Ahvaz (approval number: EE/1401.2.24.218588/scu.ac.ir).

#### 2.5.2 Carrageenan-induced murine thrombosis model

Mice were anesthetized by intraperitoneal injection of a ketamine (25mg/mL) and xylazine (5mg/mL) solution at 2mL/kg body weight. κ-Carrageenan (Sigma-Aldrich, #22048) was dissolved in sterile physiological saline at a concentration of 10 mg/mL and administered intravenously via the lateral tail vein to induce thrombosis (53). Preliminary dose-finding experiments were conducted using a range of carrageenan doses (50-400 µg per mouse) to establish a reproducible thrombosis model while minimizing systemic toxicity.

Lower doses (<100 µg) resulted in transient tail discoloration that resolved within 1–48 hours, whereas administration of 400 µg caused overt toxicity in more than half of the animals. Based on these observations, a dose of 200 µg carrageenan per mouse was selected for all subsequent experiments, as it produced stable and reproducible tail thrombosis without excessive toxicity. Following carrageenan injection, mice were returned to their cages and monitored, and thrombosis development was assessed 48 hours after administration.

#### 2.5.3 *In vivo* ITC and heparin treatments

Preliminary dose-finding and tolerability experiments were conducted to establish an appropriate *in vivo* dosing regimen for the ITC mixture. An ITC mixture stock solution (84.5 mM) was prepared and subsequently diluted for *in vivo* administration. Intraperitoneal injection of the undiluted stock solution or a 1:20 dilution caused pronounced adverse effects, including tonic spasms. Intermediate dilutions (1:40, 1:60, and 1:80) were associated with transient weakness and reduced spontaneous activity. In contrast, a 1:100 dilution was well tolerated, with no observable macroscopic pathological changes or overt behavioral abnormalities.

Based on these tolerability findings, the 1:100 dilution of the ITC mixture was selected for all subsequent *in vivo* experiments. For intraperitoneal administration, mice received 100 µL of the ITC mixture solution. For oral administration, mice received 200 µL of the same 845 µM ITC mixture solution, administered twice at 24-hour intervals. For a 25 g mouse, 100 µL of the 845 µM solution corresponds to a delivered dose of approximately 0.45 mg/kg total ITCs per intraperitoneal administration (0.13 mg/kg AITC, 0.25 mg/kg BITC and 0.07 mg/kg PEITC), and 200 µL corresponds to approximately 0.89 mg/kg per oral administration, i.e. a cumulative oral dose of approximately 1.8 mg/kg over the two administrations (**Supplementary Table S2**). The tolerability observations described above were made with solutions up to 100-fold more concentrated than the 1:100 dilution used in all reported experiments; the undiluted 84.5 mM stock corresponds to approximately 45 mg/kg total ITCs per intraperitoneal injection, which accounts for the pronounced acute effects observed at that concentration. Control animals received the corresponding diluent alone.

Heparin (Rooyan Darou Pharmaceutical Co.) was used as a reference anticoagulant and administered intraperitoneally at 2,500 U/kg, a dose widely used in experimental mouse models to achieve strong antithrombotic activity (54). Mice were randomly assigned to 11 experimental groups, as detailed in **Table 1**. The ITC mixture or heparin was administered before the carrageenan injection, at the time of the carrageenan challenge, or 1 hour after it, as specified for each group in **Table 1**. All *in vivo* analyses were performed 48 hours after carrageenan administration.

**Table 1:** Experimental treatment groups in the carrageenan-induced tail thrombosis model.

Group	Treatment and timing
T1	PBS, intravenous (i.v.)
T2	Carrageenan, intravenous (i.v.)
T3	Heparin, intraperitoneal (i.p.)
T4	Carrageenan (i.v.) + heparin (i.p.), 1-hour post-carrageenan
T5	Heparin (i.p.) + carrageenan (i.v.), 1-hour post-heparin
T6	ITC mixture, intraperitoneal (i.p.)
T7	Carrageenan (i.v.) + ITC mixture (i.p.), 1-hour post-carrageenan
T8	ITC mixture (i.p.) + carrageenan (i.v.), 1-hour post-ITC mixture
T9	ITC mixture, oral (p.o.), 0 hours and 24 hours
T10	Carrageenan (i.v.) + ITC mixture (p.o.), 0 hours and 24 hours
T11	ITC mixture (p.o.), 48 hours and 24 hours prior to carrageenan + carrageenan (i.v.)

Overview of mouse treatment groups used in the carrageenan-induced thrombosis experiments, with timing of drug administration indicated relative to carrageenan injection.

i.v., intravenous; i.p., intraperitoneal; p.o., oral administration.

#### 2.5.4 Tail thrombosis assessment

Following anesthesia, tail thrombosis was assessed 48 hours after carrageenan injection by documenting tail appearance and measuring the length of thrombosis-associated necrosis along the tail. Necrosis length was measured with a caliper by an investigator blinded to the treatment allocation, and is reported as the quantitative thrombosis endpoint of this model.

#### 2.5.5 Tail bleeding assay

Following anesthesia, 1 cm of the tail tip was amputated and immediately immersed in a tube containing pre-warmed, distilled water. Bleeding time was recorded for up to 20 minutes. Total blood loss was quantified by measuring the optical density of the collected blood at 490 nm using a microplate reader (Metertech, AccuReader M 965+). After completion of the bleeding assay, mice were euthanized by intraperitoneal injection of a ketamine (25mg/mL) and xylazine (5mg/mL) solution at 10mL/kg body weight followed by cervical dislocation, and tissues were collected for histopathological analysis.

#### 2.5.6 Histopathological evaluation

Heart, liver, lung, kidney, and tail tissues were collected and fixed in 10% buffered formalin for a minimum of 10 days. Following fixation, tissues were paraffin-embedded, sectioned at 5 µm thickness, and stained with hematoxylin and eosin (H&E) using standard histological procedures. Histopathological assessment was performed using an Olympus CX21FS1 microscope equipped with an Olympus SP-500 UZ camera. Images were acquired at 10× and 40× magnification from five consecutive microscopic fields per tissue section to evaluate structural and pathological changes. Sections were evaluated by a veterinary pathologist blinded to the treatment allocation. The histopathological assessment was descriptive by design and was intended to complement, not to replace, the quantitative tail-necrosis endpoint; no ordinal severity score was applied, and the findings summarized in **Table 2** are therefore qualitative.

**Table 2:** Histopathological summary of organ injury in mice with carrageenan-induced thrombosis following treatment with ITC mixture or heparin.

Group	Treatment	Heart	Kidney	Lung	Liver	Tail
T1	PBS	Normal	Normal	Normal	Normal	Normal
T2	Carrageenan	Myocardial necrosis	Normal	Vascular congestion with interstitial inflammatory infiltrates	Normal or hepatocellular swelling	Necrotic myositis
T3	Heparin	Myocardial hemorrhage	Normal	Congestion with hemorrhagic inflammatory infiltrates	Vascular congestion with hepatocellular swelling	Normal or hemorrhagic changes
T4	Carrageenan + heparin	Myocardial hemorrhage	Normal or glomerular congestion	Congestion with interstitial inflammatory infiltrates	Normal or hepatocellular vacuolation	Normal or focal necrosis
T5	Heparin + carrageenan	Normal or myocardial hemorrhage	Tubular degeneration	Congestion with interstitial inflammatory infiltrates	Normal or hepatocellular vacuolation	Normal
T6	ITC mixture (i.p.)	Normal	Normal or mild cell swelling	Hyperemia with interstitial inflammatory infiltrates	Normal or hepatocellular swelling	Normal
T7	Carrageenan + ITC mixture (i.p.)	Normal	Glomerular congestion	Congestion with interstitial inflammatory infiltrates	Congestion with hepatocellular swelling and vacuolation	Necrotic myositis
T8	ITC mixture (i.p.) + carrageenan	Normal	Normal or mild interstitial nephritis	Normal	Hepatocellular swelling	Normal
T9	ITC mixture (p.o.)	Normal	Normal	Normal	Hepatocellular swelling	Normal
T10	Carrageenan + ITC mixture (p.o.)	Normal	Normal or tubulo-interstitial nephritis	Normal or mild congestion	Normal	Necrotic myositis
T11	ITC mixture (p.o.) + carrageenan	Normal	Normal	Normal or mild congestion with interstitial inflammation	Normal or hepatocellular swelling	Normal

Histopathological alterations were assessed in heart, kidney, lung, liver, and tail tissues collected 48 hours after carrageenan administration. Findings are summarized qualitatively based on representative H&E-stained sections shown in **Supplementary Figure S6** (**Supporting Information**).

### 2.6 Statistical analysis

Data were analyzed using GraphPad Prism (version 10.5.0). Outliers were analyzed using the ROUT test (Q = 1%) and no outliers were found. Data distribution was assessed using the Shapiro-Wilk normality test. Unpaired Student’s t-test, or one-way ANOVA followed by Dunnett’s *post hoc* test, was applied as appropriate. For time course experiments, a two-way repeated-measures ANOVA followed by Tukey’s multiple comparisons test was performed.

Data are presented as mean ± SEM, as indicated in the figure legends. Statistical significance is denoted as *p < 0.05; **p < 0.005; ***p < 0.0005; ****p < 0.0001. All experiments were performed with at least 3 independent biological replicates. Sample sizes are specified in **Supplementary Table S1**.

## 3 Results

### 3.1 A defined ITC mixture inhibits platelet aggregation across diverse agonists

We began by testing how different concentrations of a defined combination of AITC, BITC, and PEITC (hereafter referred to as the ITC mixture) affect TRAP-induced aggregation and platelet activation. The experimental plan is depicted in **Figure 1A**. At lower doses (84.5-422 µM) inhibition remained incomplete, while increasing concentrations progressively reduced TRAP-driven aggregation (**Figures 1B, C**). However, the highest concentration tested (2530 µM) produced a strong increase in Annexin V binding, consistent with cytotoxicity (**Figure 1D**). In contrast, the defined ITC mixture at a total concentration of 845 µM (320 µM AITC, 422.5 µM BITC, and 102.5 µM PEITC) blocked TRAP-induced aggregation without elevating apoptotic markers. This concentration was therefore used for all subsequent experiments. Plasma-membrane integrity was likewise preserved at this concentration: LDH release at 845 µM did not differ from vehicle control at any of the exposure intervals used in this study, whereas 2530 µM produced a significant increase (**Supplementary Figure S1**, **Supporting Information**).

**Figure 1:**
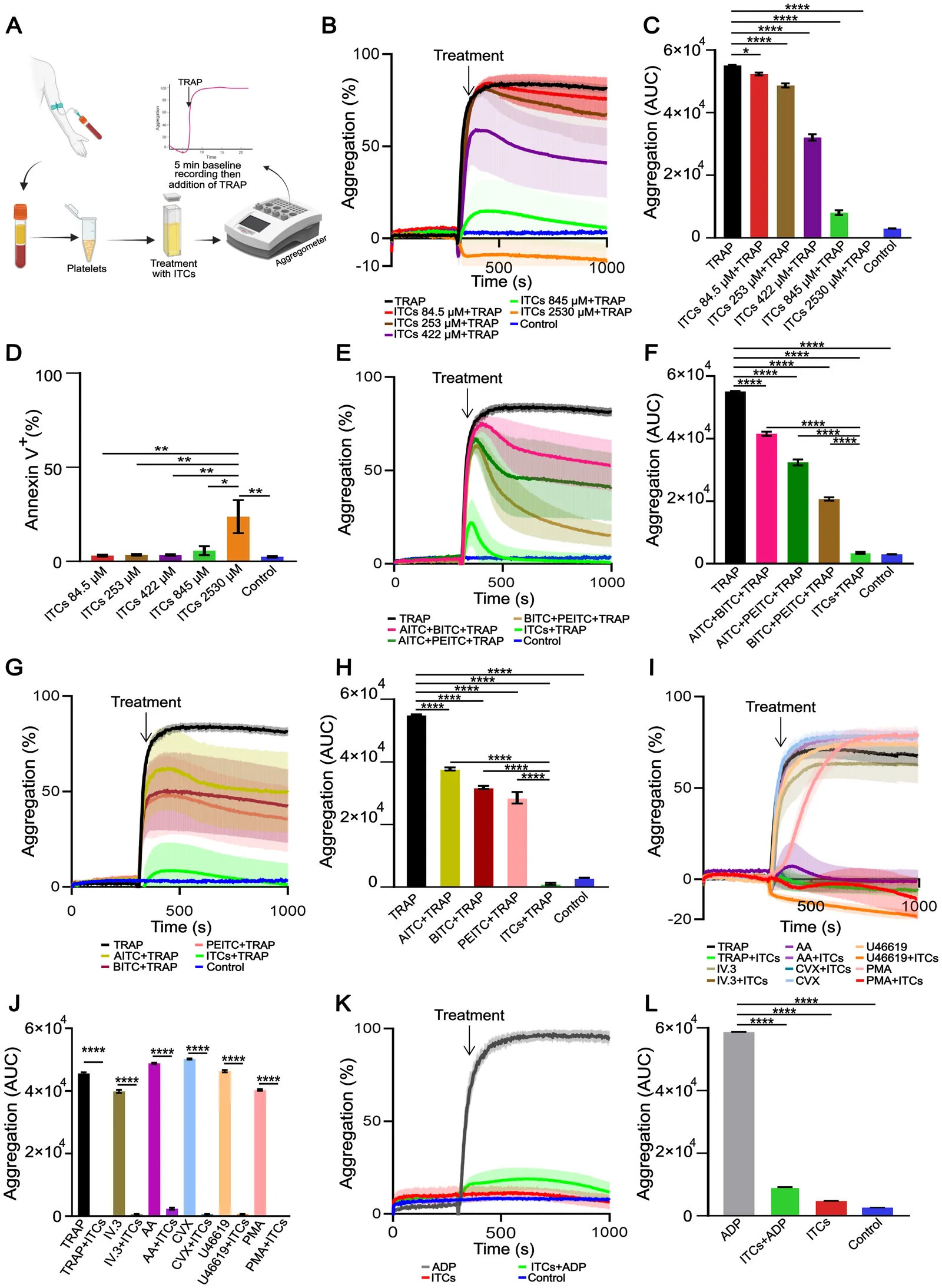
A defined ITC mixture suppresses human platelet aggregation across multiple activation pathways. “ITCs” refers to the defined mixture of AITC, BITC, and PEITC. **(A)** Schematic of the experimental workflow for platelet pretreatment with ITCs. **(B)** Representative light transmission aggregometry traces of TRAP (25 µM)-stimulated platelets pretreated for 5 minutes with increasing concentrations of ITCs (0, 84.5, 253, 422, 845, or 2530 µM). **(C)** Maximal aggregation and area under the curve (AUC) corresponding to **(B)**. **(D)** Annexin V binding after 5 minutes ITC exposure, showing that lower doses of the ITC mixture do not increase apoptotic markers. **(E)** Representative light transmission aggregometry traces of TRAP (25 µM)-stimulated platelets pretreated for 5 minutes with the ITC mixture (845 µM total concentration) or with pairwise ITC combinations at equivalent total concentrations. **(F)** Maximal aggregation and area under the curve (AUC) corresponding to **(E)**. **(G)** Representative light transmission aggregometry traces of TRAP (25 µM)-stimulated platelets pretreated for 5 minutes with ITCs (845 µM), or individual ITCs (845 µM each). **(H)** Maximal aggregation and area under the curve (AUC) corresponding to **(G)**, showing that, at the tested concentration, PEITC produced the strongest inhibition among the individual compounds, while AITC produced the weakest; none matched the full ITC mixture. **(I)** Representative light transmission aggregometry traces of AA-, U46619-, CVX-, IV.3-, or PMA-stimulated platelets pretreated for 5 minutes with ITCs (845 µM). **(J)** Maximal aggregation and area under the curve (AUC) corresponding to **(I)**. **(K)** Representative light transmission aggregometry traces of ADP (100 µM)-stimulated PRP pretreated for 5 minutes with ITCs (845 µM). **(L)** Quantification of maximal aggregation and area under the curve (AUC) corresponding to the experiments shown in **(K)**. Data represent mean ± SEM from at least 3 independent biological replicates, as detailed in **Supplementary Table S1**. Statistics were performed by one-way ANOVA with Dunnett’s *post hoc* test (*p < 0.05; **p < 0.005; ****p <0.0001).

We then explored whether this pronounced inhibitory effect depended on cooperation among the three ITCs or if it could be achieved with two-component combinations (**Figures 1E, F**) or individual compounds (**Figures 1G, H**). In these experiments the single agents and the pairwise combinations were applied at the same total concentration of 845 µM as the complete mixture, rather than at equipotent concentrations. Under these conditions, neither the individual compounds nor any two-component combination reproduced the full suppression observed with the complete mixture (**Figures 1E–H**). Additional isothiocyanates, including sulforaphane isothiocyanate (SFN) and indole-3-isothiocyanate (IITC), were initially evaluated. However, these compounds demonstrated substantially weaker inhibitory activity compared to the isothiocyanates included in the mixture and were consequently excluded from further analyses (**Supplementary Figure S3**, **Supporting Information**). Because a fixed-ratio dilution series and a formal isobolographic or combination-index analysis were not performed, these data show that the three-component mixture is more effective than its individual components or the pairwise combinations tested at a matched total concentration, but they do not formally demonstrate pharmacological synergy.

To determine whether this inhibitory behavior was confined to TRAP signaling or reflected a broader effect, we next evaluated the ITC mixture across several well-established platelet agonists and investigated its ability to blunt secondary amplification mechanisms. Pre-exposure to the ITC mixture for 5 minutes before stimulation sharply dampened aggregation induced by arachidonic acid, U46619, convulxin, FcγRIIa crosslinking by IV.3, and PMA, demonstrating its ability to interfere with pathways driven by cyclooxygenase, thromboxane receptors, GPVI, FcγRIIa, and PKC (**Figures 1I, J**). The ITC mixture also reduced aggregation triggered by ADP, a major autocrine and paracrine mediator of platelet recruitment (**Figures 1K, L**). This finding shows disruption of downstream reinforcement loops, even when activation relies on secondary agonists rather than primary receptor engagement. Taken together, these results show that the ITC mixture acts as a broad and multimodal inhibitor of platelet activation and aggregation, blocking responses to a range of primary agonists and suppressing essential amplification pathways that sustain thrombus growth.

### 3.2 The ITC mixture disrupts early platelet aggregation but does not reverse degranulation or surface marker exposure after initiation

To examine whether the ITC mixture can affect platelet aggregates after their formation, platelets were stimulated with TRAP, and the ITC mixture was added either 25 s or 300 s later (as outlined in **Figure 2A**). Addition of the ITC mixture at 25 s led to a clear reduction in ongoing aggregation, whereas addition at 300 s produced only a gradual fall in light transmission. These findings indicate that the ability of the ITC mixture to interfere with aggregation diminishes as activation progresses (**Figure 2B**). A comparable time-dependent effect was observed with other platelet agonists, including arachidonic acid, U46619, convulxin, IV.3, and PMA (**Supplementary Figure S4**, **Supporting Information**). Light transmission aggregometry reports the bulk optical density of the stirred suspension, and a fall in the trace after stimulation cannot by itself distinguish physical disassembly of aggregates from loss of single platelets from aggregates or from changes in platelet shape and light-scattering properties. The aggregometry data are therefore interpreted here as evidence of aggregate destabilization; orthogonal confirmation of true disassembly by imaging, flow-based measurement of aggregate size, clot retraction or perfusion assays was not performed in this study.

**Figure 2:**
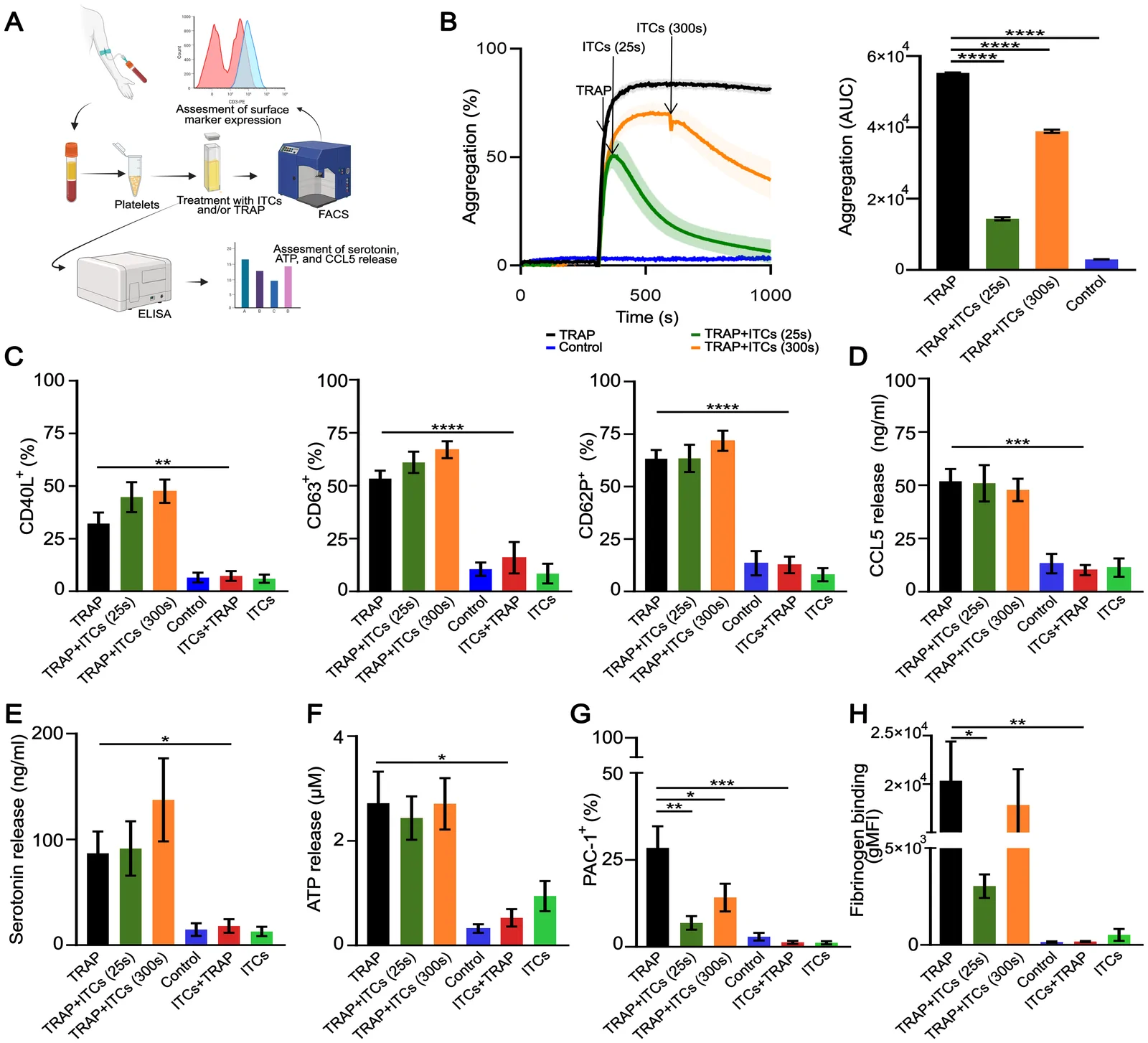
Effects of delayed ITC addition on platelet aggregation, granule secretion, and integrin activation. “ITCs” refers to the defined mixture of AITC, BITC, and PEITC. **(A)** Schematic of the experimental workflow used to assess the effects of delayed ITC addition on platelet activation, granule secretion, and integrin activation. **(B)** Representative light transmission aggregometry traces of TRAP-induced platelet aggregation, with ITCs (845 µM) added at defined time points: at 25 s or 300 s after TRAP addition. **(C)** Surface expression of CD40L, CD63, and CD62P, assessed by flow cytometry. **(D)** CCL5 release measured by ELISA as an index of α-granule secretion. **(E)** Serotonin release and **(F)** ATP release, measured as indices of dense-granule secretion. **(G)** PAC-1 binding assessed by flow cytometry as a readout of integrin αIIbβ3 activation. **(H)** FITC-fibrinogen binding measured by flow cytometry as an additional readout of integrin αIIbβ3 activation. Data represent mean ± SEM from at least 3 independent biological replicates, as detailed in **Supplementary Table S1**. Statistical significance was determined by one-way ANOVA with Dunnett’s *post hoc* test: *p < 0.05; **p < 0.005; ***p < 0.0005; ****p < 0.0001. Created in BioRender. Hachani, K. (2026) https://BioRender.com/aplao9v

To further examine this, we evaluated whether the ITC mixture could reverse the surface exposure of activation-dependent markers once initiated. ITC pre-treatment prevented TRAP-induced upregulation of CD62P, CD63, and CD40L. In contrast, addition of the mixture at either 25 s or 300 s after stimulation did not reduce these surface marker levels (**Figure 2C**). Consistent results were obtained when platelets were stimulated with other physiological agonists, demonstrating that the time-dependent loss of ITC mixture efficacy is not restricted to TRAP-mediated activation (**Supplementary Figure S5**, **Supporting Information**). Our quantification of α-granule release (CCL5) and dense-granule release (ATP and serotonin) further indicated that this lack of reversibility extends to granule secretion. ITC pre-treatment markedly suppressed TRAP-induced secretion, whereas post-treatment at either time point did not reduce the concentrations of released mediators detected in the supernatant (**Figures 2D–F**). These data indicate that granule exocytosis is not reversed by the ITC mixture once initiated. Finally, we assessed the effects of delayed ITC mixture addition on integrin αIIbβ3 activation. Pre-treatment strongly reduced PAC-1 binding and fibrinogen association. When the ITC mixture was added 25 s after TRAP stimulation, both readouts were partially reduced, yet addition at 300 s resulted in only a modest decrease in PAC-1 binding and no detectable change in fibrinogen binding (**Figures 2G, H**). Together, these data indicate that the ITC mixture can interfere with early aggregation and integrin activation shortly after platelet stimulation but does not reverse established platelet activation or stable integrin engagement.

### 3.3 Kinome profiling predicts widespread signaling suppression and confirms reduced AKT phosphorylation

To identify signaling pathways targeted by the ITC mixture, we performed unbiased kinase activity profiling using the PamGene platform. As reflected in the kinase activity profiles (**Figures 3A, B**), ITC mixture exposure was associated with a widespread reduction in peptide phosphorylation events linked to platelet activation across conditions. In unstimulated platelets, treatment with the ITC mixture was associated with reduced predicted basal activity across multiple signaling modules, including receptor tyrosine kinase pathways (such as VEGFR2 and ERBB family members), MAPK1/3 (ERK1/2), PI3K/AKT, and Gα-mediated signaling (**Figure 3C**). A more pronounced reduction in predicted kinase activity was apparent in platelets receiving ITC pre-treatment and TRAP stimulation. In this setting, pathways downstream of receptor engagement, including PLC-dependent calcium signaling, Rho GTPase-NADPH oxidase pathways, and DAG/IP_3_-driven secondary messenger amplification, were predicted to be attenuated (**Figure 3C**). The affected nodes are those that integrate calcium flux, redox regulation and metabolic control, which is consistent with a reduced capacity to sustain activation. The PamGene platform measures phosphorylation of peptide substrates immobilized on the array and infers upstream kinase activity from these signals; the pathway-level directionality reported here is therefore a prediction rather than a direct measurement of kinase activity in the platelet.

**Figure 3:**
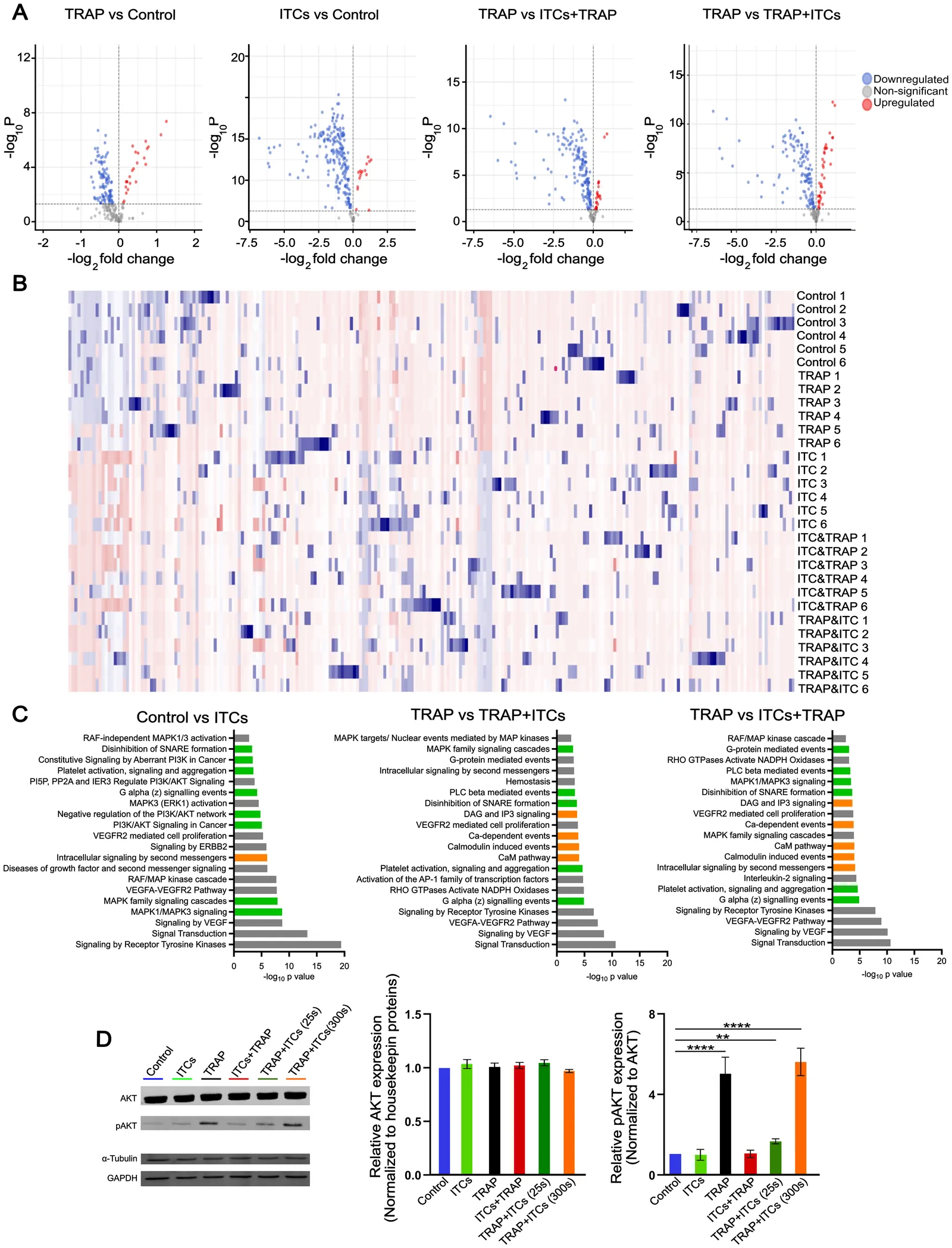
ITC mixture is associated with predicted suppression of platelet signaling networks and with attenuated AKT activation. “ITCs” refers to the defined mixture of AITC, BITC, and PEITC. **(A)** Volcano plots summarizing differential phosphorylation derived from PamGene kinome profiling. Plots show log_2_ fold change versus −log_10_ adjusted *p* value for the indicated comparisons (TRAP vs control, ITC vs control, TRAP vs TRAP+ITC, and TRAP vs ITC+TRAP), based on merged serine/threonine kinase (STK) and protein tyrosine kinase (PTK) datasets. Each point represents an individual phosphosite. Phosphosites with *p* < 0.05 and positive log_2_ fold change are shown in red (upregulated), those with *p* < 0.05 and negative log_2_ fold change in blue (downregulated), and nonsignificant sites are shown in gray. Horizontal dashed lines indicate the *p* = 0.05 threshold, and vertical dashed lines indicate a log_2_ fold change of ±1. **(B)** Heatmaps depicting global kinase activity profiles across experimental conditions. Relative kinase activity (z-scores) for STKs and PTKs is shown for control (300 s), TRAP-stimulated platelets (300 s), and platelets treated with ITCs either prior to TRAP stimulation (300 s ITC pre-treatment followed by 300 s TRAP stimulation) or 25 s after TRAP stimulation (325 s TRAP stimulation, with the ITC mixture added 25 s after TRAP). The color scale ranges from blue (lower activity) to red (higher activity). Experimental conditions are indicated by the row annotation bar. **(C)** Pathway-level enrichment analysis summarizing kinome alterations across conditions. Bar plots show regulated signaling pathways, including receptor tyrosine kinase signaling (e.g., VEGFR2 and EGFR family members), PI3K/AKT, MAPK/ERK, PLC-mediated Ca^2+^ signaling, Rho GTPase pathways, and secondary messenger systems (DAG/PKC and IP_3_/Ca^2+^). Bar length represents −log_10_ *p* value, indicating pathway significance. Orange indicates pathways predicted to be activated, green indicates inhibited, and gray indicates significantly enriched pathways without a consistent activation or inhibition direction. **(D)** Immunoblot analysis of AKT signaling in human platelets. Representative blots show phosphorylation of AKT at Ser473 and total AKT under the indicated conditions. Right panels show densitometric quantification normalized to both GAPDH and α-tubulin, and expressed relative to control. Data represent mean ± SEM from at least 3 independent biological replicates, as detailed in **Supplementary Table S1**. Statistical significance was assessed by one-way ANOVA with Dunnett’s multiple comparisons test (**p < 0.005, ****p < 0.0001).

When the ITC mixture was added 25 s after TRAP stimulation, mimicking intervention after signaling initiation, the kinome profile revealed only partial attenuation. Predicted activity within pathways such as VEGF-VEGFR2, Gαz, Rho-NOX, and MAPK was reduced relative to TRAP alone, whereas other signaling axes remained largely active (**Figure 3C**). This pattern suggests that the ITC mixture attenuates ongoing signaling less effectively once phosphorylation cascades are established.

To validate PI3K/AKT signaling as a key pathway identified by kinome analysis, we assessed AKT phosphorylation as a representative pathway readout. TRAP stimulation induced robust phosphorylation of AKT at Ser473. While this response was completely abolished when ITCs were applied as a pre-treatment, it was only partially reversed when applied 25 s after TRAP stimulation, without affecting total AKT expression. In contrast, ITC addition at 300 s failed to reverse AKT phosphorylation (**Figure 3D**). These results show that the ITC mixture reduces AKT phosphorylation, and the kinome analysis suggested attenuation of receptor-associated inputs, including receptor tyrosine kinase- and G protein-mediated signaling, together with downstream effectors involved in calcium handling and redox-sensitive pathways. This coordinated disruption of redox balance and metabolic function is consistent with reduced energetic support for sustained platelet activation.

### 3.4 The ITC mixture induces oxidative stress and mitochondrial dysfunction in platelets

Given the strong effects of the ITC mixture on signaling pathways linked to calcium handling, redox regulation, and metabolism, we next examined impacts on intracellular Ca^2+^ dynamics, the intracellular oxidant signal, and mitochondrial function. Intracellular Ca^2+^ flux was monitored using the fluorescent indicator Calbryte-520AM. Resting platelets displayed low basal Ca^2+^ levels, which increased rapidly upon TRAP stimulation, consistent with platelet activation (**Figure 4A**). Treatment with the ITC mixture slightly elevated basal Ca^2+^ levels.

**Figure 4:**
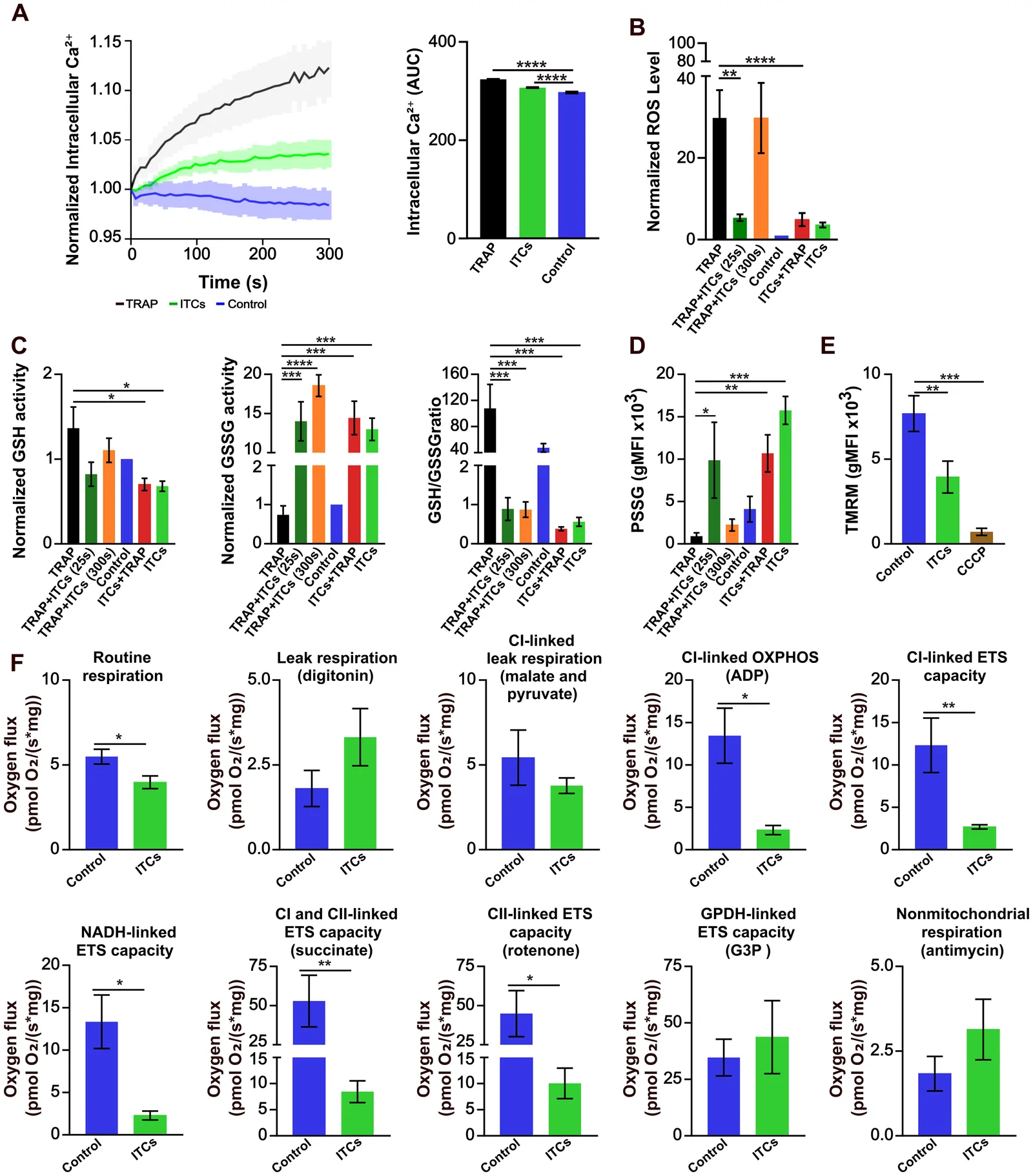
ITC mixture disrupts platelet Ca^2+^ signaling, redox homeostasis, and mitochondrial function. “ITC” refers to the defined mixture of AITC, BITC, and PEITC. **(A)** Intracellular Ca^2+^ mobilization measured using Calbryte-520AM. TRAP stimulation induces a rapid Ca^2+^ rise, whereas ITC treatment modestly elevates baseline Ca^2+^ levels. **(B)** CellROX™ fluorescence used as an intracellular oxidant readout. TRAP increases the signal. ITC pre-treatment maintains it near baseline, and ITC addition 25 s after TRAP limits further accumulation compared with TRAP alone, but delayed ITC addition does not reduce the elevated CellROX signal. **(C)** Glutathione measurements show reduced glutathione (GSH), oxidized glutathione (GSSG), and the GSH/GSSG ratio. ITC treatment is associated with increased GSSG and a reduced GSH/GSSG ratio. **(D)** Protein S-glutathionylation (PSSG) assessed by flow cytometry. ITC treatment, applied either alone, before, or shortly after TRAP stimulation, increases PSSG levels, while delayed ITC addition produces minimal change. **(E)** Δψm measured using TMRM, CCCP was used as a positive control to dissipate Δψm. **(F)** Mitochondrial respiration parameters derived from high-resolution respirometry. 845 µM ITC mixture treatment reduces routine respiration, complex I (CI)-linked OXPHOS capacity (ADP), CI-linked ETS capacity (CCCP), NADH-linked ETS capacity (glutamate), convergent CI- and CII-linked ETS capacity (succinate) and CII-linked ETS capacity (rotenone), whereas leak respiration (digitonin), CI-linked leak respiration (malate and pyruvate), GPDH-linked ETS capacity (glycerol-3-phosphate) and non-mitochondrial oxygen consumption (antimycin A) remain unchanged. Data represent mean ± SEM from at least 3 independent biological replicates, as detailed in **Supplementary Table S1**. Statistical significance was determined using unpaired Student’s *t*-test or one-way ANOVA with Dunnett’s multiple comparisons test, as indicated: *p ≤ 0.05; **p ≤ 0.005; ***p ≤ 0.0005; ****p ≤ 0.0001.

We next assessed the intracellular oxidant signal using CellROX™ Green fluorescence (**Figure 4B**). TRAP stimulation caused a pronounced increase in CellROX fluorescence. ITC pre-treatment reduced this response to the level seen with the ITC mixture alone, which was itself approximately fourfold above vehicle control. When the ITC mixture was added 25 s after TRAP stimulation, the signal declined toward baseline. Addition at 300 s did not reduce the elevated signal.

These findings are consistent with suppression of the early activation-associated oxidant signal and with a loss of efficacy once oxidative signaling has been established. This readout however, should be interpreted with caution as retention of CellROX Green is influenced by the mitochondrial membrane potential. The reduced CellROX signal in ITC-treated platelets can therefore not be attributed unambiguously to a reduction in ROS production, and this readout is not independent of the mitochondrial phenotype. We therefore repeated these measurements using CellROX™ Orange, a cytosolic probe that, unlike CellROX™ Green, does not accumulate in the nucleus or in mitochondria and whose retention is consequently not contingent on Δψm (**Supplementary Figure S2**, **Supporting Information**). The pattern was reproduced in full. TRAP increased the oxidant signal significantly over vehicle control. ITC pre-treatment held the signal at the level produced by the mixture alone, addition of the mixture 25 s after TRAP returned it to a comparable level, and addition at 300 s left it indistinguishable from TRAP alone. The reduction in the activation-associated oxidant signal is therefore unlikely to be explained by the loss of Δψm alone. The concurrent rise in GSSG and in protein S-glutathionylation (**Figures 4C, D**) indicates that the intracellular redox balance is in fact shifted toward oxidation, and a Δψm-independent oxidant probe was therefore used to resolve this apparent discrepancy.

To further characterize the effect of the ITC mixture on redox regulation, intracellular glutathione pools were quantified. ITC treatment increased oxidized glutathione (GSSG) levels under all conditions tested, resulting in a consistently reduced GSH/GSSG ratio (**Figure 4C**). This shift toward a more oxidized intracellular state was accompanied by increased protein S-glutathionylation (PSSG) levels, selectively observed in platelets treated with the ITC mixture alone, ITC pre-treatment, or early post-activation ITC addition (25 s). In contrast, delayed ITC mixture addition at 300 s did not increase PSSG levels, indicating that ITC mixture-induced protein S-glutathionylation is restricted to early phases of platelet activation (**Figure 4D**). Because calcium and redox signals converge on mitochondrial function, we next examined the impact of the ITC mixture on platelet mitochondrial integrity and bioenergetics. Assessment of Δψm using TMRM revealed a marked reduction in Δψm in ITC-treated platelets, consistent with partial mitochondrial depolarization relative to controls and approaching the level induced by the mitochondrial uncoupler CCCP (**Figure 4E**).

To further define the functional consequences of this depolarization, mitochondrial respiration was analyzed via high-resolution respirometry using the Oxygraph-2k system (**Figure 4F**). ITC mixture treatment resulted in a broad but selective suppression of mitochondrial oxygen consumption: routine respiration of intact platelets was reduced, and ADP-stimulated complex I (CI)-linked OXPHOS capacity, CI-linked ETS capacity and NADH-linked ETS capacity were all impaired. Convergent CI- and CII-linked ETS capacity (succinate) and CII-linked ETS capacity (rotenone) were likewise reduced. In contrast, leak respiration after digitonin permeabilization, CI-linked leak respiration (malate and pyruvate), GPDH-linked ETS capacity (glycerol-3-phosphate) and non-mitochondrial oxygen consumption (antimycin A) were unchanged, indicating the ITC mixture preferentially compromises mitochondrial respiratory capacity rather than extra-mitochondrial oxygen-consuming processes. These changes occurred without loss of plasma-membrane integrity: LDH release at 845 µM was indistinguishable from vehicle control after 300, 900 and 1800 s of exposure, that is, across the full duration of the immunoblotting and respirometry protocols, whereas the cytotoxic concentration of 2530 µM produced a clear increase (**Supplementary Figure S1**, **Supporting Information**). These observations point to a shift toward an oxidized and energetically compromised platelet state following exposure to the ITC mixture, characterized by attenuated calcium signaling, restricted early ROS amplification, and impaired mitochondrial bioenergetic capacity. Collectively, these findings define an early redox shift characterized by increased GSSG, temporally restricted PSSG, attenuated Ca^2+^ signaling, and impaired mitochondrial respiration.

### 3.5 The ITC mixture disrupts mitochondrial cristae architecture while preserving organelle size

To assess whether the ITC mixture alters platelet mitochondrial ultrastructure, platelets were examined using TEM (**Figure 5A**). The mitochondrial cross-sectional area was comparable between control and ITC-treated platelets (**Figure 5B**), indicating that ITC treatment does not induce mitochondrial swelling or major changes in organelle size under these experimental conditions. Qualitative analysis of TEM images indicated differences in inner mitochondrial membrane organization between conditions (**Figure 5A**). To quantify these alterations, cristae architecture was measured. Exposure to the ITC mixture led to a reduction in the number of intact cristae per mitochondrion (**Figure 5C**). Additionally, the proportion of mitochondria exhibiting severe cristae loss, defined as one or fewer identifiable crista, was substantially higher in ITC-treated platelets compared with controls (**Figure 5D**). Collectively, these findings demonstrate that the ITC mixture selectively disrupts inner mitochondrial membrane organization without altering overall mitochondrial size.

**Figure 5:**
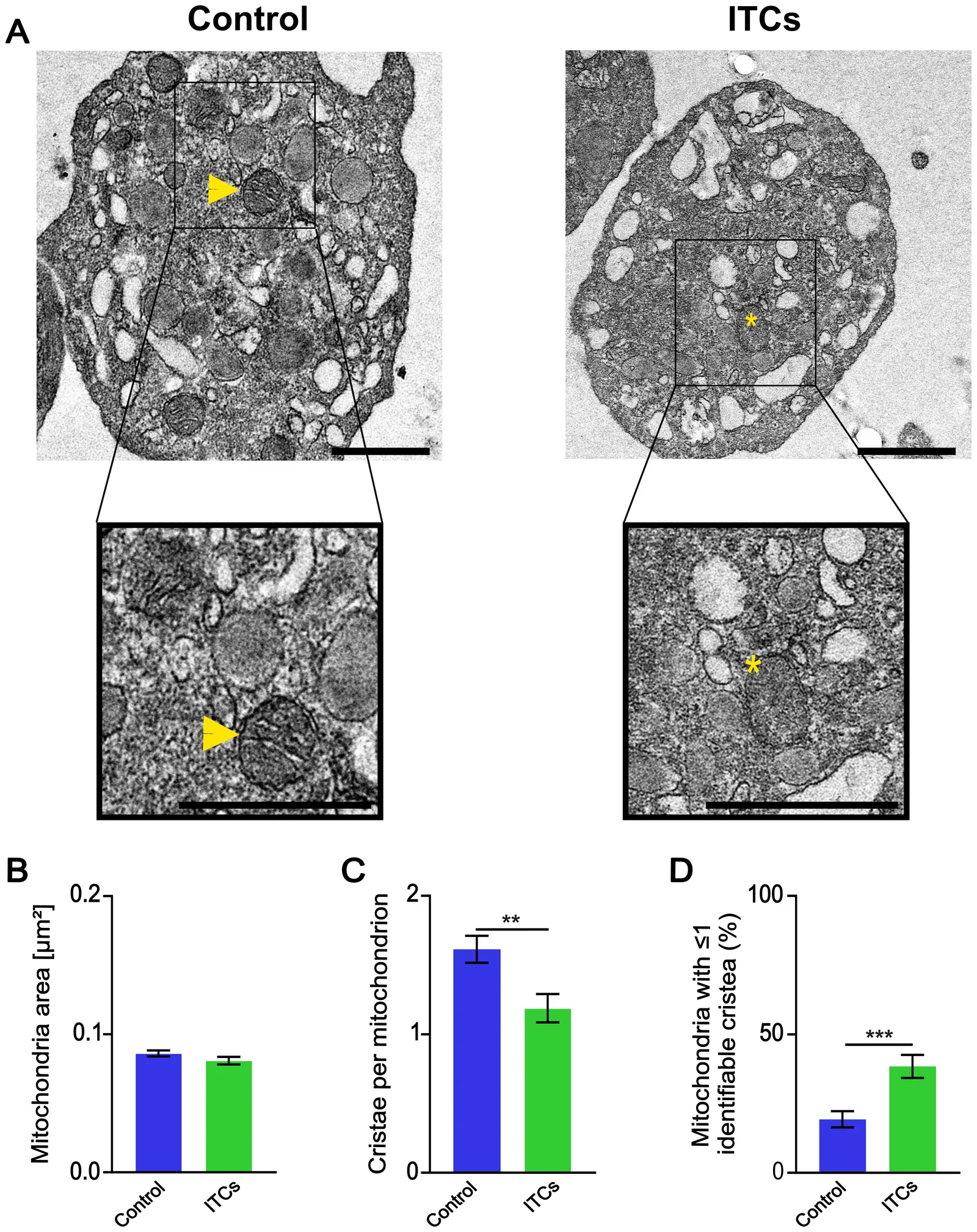
ITC mixture disrupts mitochondrial cristae architecture in platelets without altering organelle size. “ITC(s)” refers to the defined mixture of AITC, BITC, and PEITC. **(A)** Representative transmission electron microscopy images of platelets from control and ITC-treated samples (845 µM, 300 s). Control platelets display mitochondria with densely packed, well-organized cristae, whereas ITC-treated platelets frequently exhibit mitochondria with disorganized or fragmented, or poorly defined cristae, despite comparable overall mitochondrial shape and size. Insets show higher-magnification views, highlighting cristae alterations (arrowheads). Scale bars are 1 µm. **(B)** Quantification of mitochondrial cross-sectional area reveals no difference between control and ITC-treated platelets. **(C)** The average number of intact cristae per mitochondrion is reduced following ITC treatment. **(D)** The proportion of mitochondria lacking structured cristae (defined as ≤1 identifiable crista) is markedly increased in ITC-treated platelets. Data are presented as mean ± SEM and were derived from at least 50 mitochondria per donor (at least 3 independent biological replicates, as detailed in **Supplementary Table S1**). Statistical significance was assessed using unpaired Student’s *t*-test and is indicated as, **p ≤ 0.005, ***p ≤ 0.0005.

### 3.6 The ITC mixture reduces carrageenan-induced thrombosis and prolongs bleeding *in vivo*

To determine whether the antiplatelet effects of the ITC mixture could offer protection against thrombosis *in vivo*, we employed a carrageenan-induced tail thrombosis model that produces systemic inflammation and reproducible thrombus formation (**Figure 6A**). The experimental groups, dosing regimens, and routes of administration are summarized in **Table 1**. The ITC mixture was administered before the carrageenan challenge, at the time of challenge (oral groups, 0 and 24 hours), or 1 hour after challenge (intraperitoneal groups), via intraperitoneal or oral routes as specified in **Table 1**. Heparin was included as a reference anticoagulant, and carrageenan-only mice served as controls.

**Figure 6:**
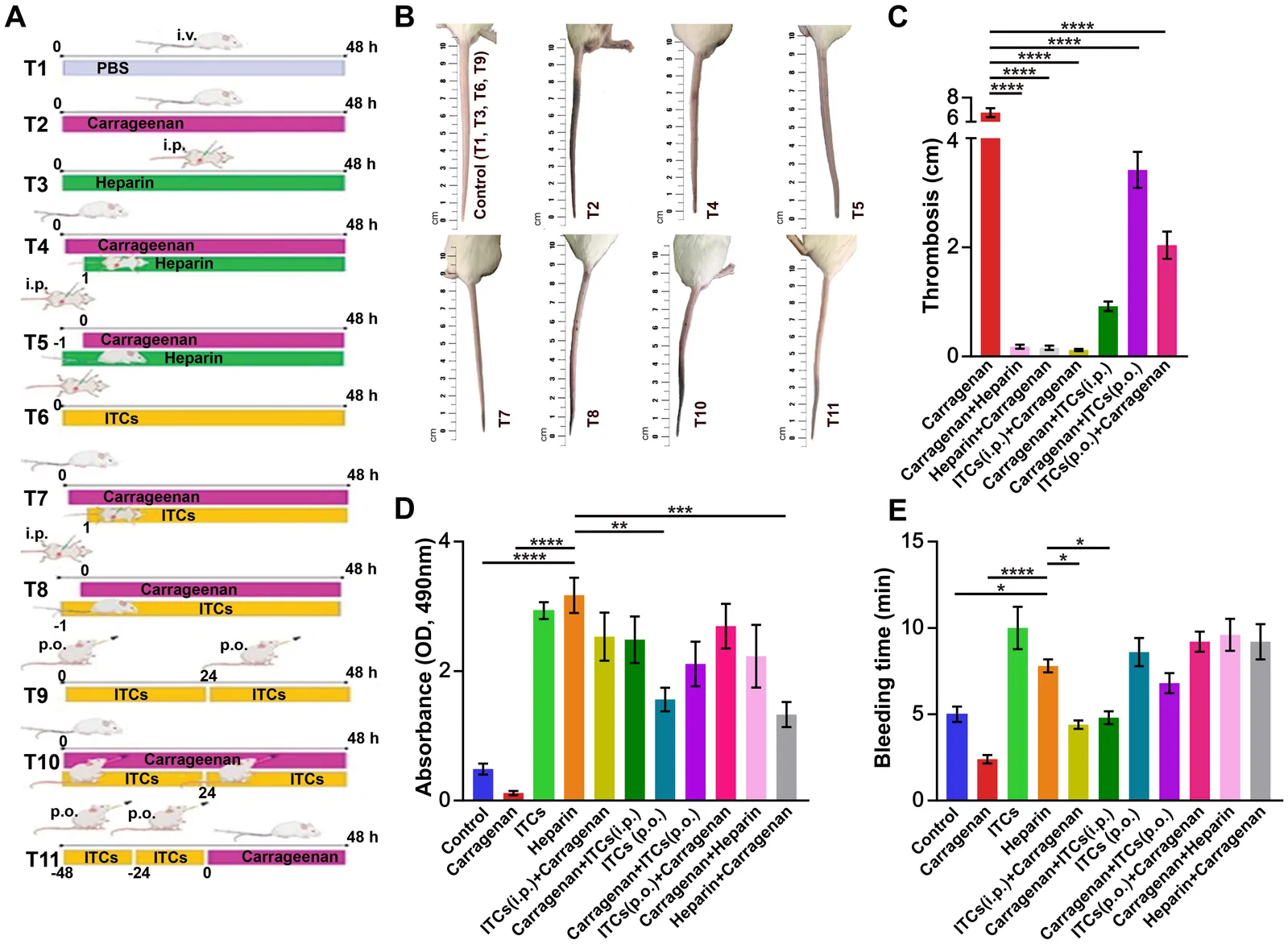
The ITC mixture attenuates thrombosis in a carrageenan-induced mouse model, with heparin as a reference anticoagulant. **(A)** Schematic overview of the *in vivo* thrombosis experimental design. Mice received κ-carrageenan (200 µg, i.v.) to induce systemic inflammation and tail vein thrombosis. The ITC mixture (845 µM) was administered by the intraperitoneal route 1 hour before or 1 hour after carrageenan, or by the oral route at the time of challenge (0 and 24 hours) or before challenge (48 and 24 hours prior), as specified in **Table 1**. Heparin (2500 U/kg, i.p.) served as a reference anticoagulant control. **(B)** Thrombotic outcomes were assessed 48 hours after carrageenan administration. **(C)** Quantification of tail thrombosis severity based on tail necrosis length. **(D)** Total blood loss measured in the tail-bleeding assay, quantified by hemoglobin absorbance at 490 nm for carrageenan-treated mice receiving ITC mixture via i.p. or p.o. administration. **(E)** Bleeding time following standardized tail transection. Both ITC mixture- and heparin-treated mice displayed prolonged bleeding times compared with carrageenan-only controls. Data represent mean ± SEM from at least 3 independent biological replicates, as detailed in **Supplementary Table S1**. Statistical significance was assessed using one-way ANOVA with Dunnett’s multiple comparisons test: *p ≤ 0.05; **p ≤ 0.005; ***p ≤ 0.0005; ****p ≤ 0.0001.

In control group, carrageenan induced pronounced tail vein thrombosis accompanied by distal tail necrosis (**Figure 6B**). In contrast, mice receiving the ITC mixture, across the tested administration routes and treatment schedules, exhibited a substantial reduction in thrombotic tail injury. Across all groups treated with the ITC mixture, tail necrosis was consistently reduced, often being absent or limited to minor distal changes, with an overall pattern comparable to that observed in heparin-treated controls. When administered 1 hour after the carrageenan challenge, the ITC mixture limited the progression of thrombosis, indicating that it retains antithrombotic activity when given after the challenge (**Figure 6C**).

Because antithrombotic efficacy is frequently associated with altered hemostasis, we next assessed bleeding parameters. Compared with carrageenan-only controls, ITC mixture-treated mice displayed increased blood loss, quantified by hemoglobin absorbance in the bleeding assay, and prolonged tail bleeding time (**Figures 6D, E**). Notably, despite prolonged bleeding parameters, no overt hemorrhagic complications were detected in mice receiving the ITC mixture.

Collectively, these data demonstrate that the ITC mixture exerts robust antithrombotic effects *in vivo*, reducing carrageenan-induced thrombosis when administered before, at the time of, or after the carrageenan challenge. At the doses and treatment schedules tested (**Table 1**), heparin was included as a reference anticoagulant, and ITC treatment reduced tail-necrosis length and altered bleeding parameters to a similar extent under the conditions of this model.

### 3.7 The ITC mixture mitigates carrageenan-induced organ injury in histopathological analyses

To determine whether the antithrombotic effects of the ITC mixture extend beyond the tail and confer protection against systemic tissue injury, we performed histopathological analyses of major organs 48 hours after carrageenan administration (**Supplementary Figure S6**, **Supporting Information**). Tissue alterations across all experimental groups are summarized in **Table 2**. As expected, carrageenan alone induced widespread pathological changes consistent with systemic thrombosis and inflammation. Histological examination revealed frequent thrombotic lesions in the heart and pulmonary vasculature, accompanied by pulmonary congestion and interstitial inflammatory changes. In addition, liver sections showed hepatocellular swelling and vascular congestion, while renal tissue exhibited signs of tubular and interstitial involvement in some subjects. Tail tissue displayed pronounced necrosis and hemorrhage, consistent with severe local thrombosis. Treatment with heparin mitigated many carrageenan-induced lesions, markedly reducing thrombus burden in the heart and lungs and limiting tail muscle necrosis. However, residual pathological changes were still observed in some subjects; this included focal hemorrhage, vascular congestion, and pulmonary thromboembolic features. These findings were consistent with the anticoagulant activity and bleeding liability effects of heparin and confirmed the sensitivity of this model to standard antithrombotic intervention.

In contrast, mice treated with the ITC mixture showed substantially improved histological outcomes across multiple organs. Whether given before the carrageenan challenge, at the time of challenge, or 1 hour after it, and by either route, ITC mixture treatment was associated with a marked reduction in cardiac thrombus formation and pulmonary microthrombi. Reduced cardiac and pulmonary thrombotic lesions and reduced tail tissue injury were also seen after oral administration. Overall, the descriptive histopathological findings were consistent with reduced carrageenan-associated tissue injury in the ITC-treated groups and complemented the quantitative tail-necrosis measurements. The effect was seen with both routes of administration, suggesting a systemic antithrombotic effect *in vivo*. As observed with heparin, ITC mixture treatment also altered bleeding parameters.

## 4 Discussion

Platelet activation and aggregation are central drivers of arterial thrombosis and contribute substantially to venous thrombosis and inflammation-associated coagulopathies. These processes significantly contribute to morbidity and mortality associated with cardiovascular disease globally, necessitating antithrombotic therapies that inhibit thrombus formation without increasing bleeding risk. In this study, we investigated a nutraceutical-inspired strategy to modulate platelet function using naturally derived isothiocyanates. Specifically, we examined a defined ITC mixture composed of AITC, BITC, and PEITC, the principal active components of the phytotherapeutic preparation ANGOCIN^®^ *Anti-Infekt N*, which is clinically approved for antimicrobial indications, but is not currently used for platelet inhibition or antithrombotic therapy. The ITC mixture was shown, both *in vitro* and *in vivo*, to suppress initial platelet activation and aggregation, and to interfere with platelet aggregate formation. Together, these findings provide mechanistic insight into how the ITC mixture regulates platelet responses and supports its potential as a foundation for novel antithrombotic strategies.

A central observation of this work is that, at a matched total concentration, the three-component ITC mixture produced greater inhibition than its individual constituents. While previous studies have reported antiplatelet effects of single ITCs, such as SFN and AITC, often at relatively high concentrations (39, 41), we found that neither AITC, BITC, PEITC, nor any pairwise combination reproduced the magnitude of inhibition observed with the complete mixture under the tested conditions. While all ITCs share the reactive -N=C=S electrophilic group, their side-chain structures differ substantially, influencing membrane permeability, intracellular retention, and thiol reactivity (55). BITC and PEITC, which contain more hydrophobic aromatic moieties (55), are likely to penetrate platelets more efficiently and persist longer intracellularly than the smaller, more volatile AITC. At the tested concentration, PEITC produced the strongest inhibitory effect among the individual compounds, whereas AITC produced the weakest. These findings align with reports that ITCs bearing longer or more hydrophobic side chains display enhanced biological activity (56). PEITC represents approximately 12% of the mixture. Because single agents and the mixture were compared at equivalent total concentration rather than at equipotent concentrations, and no fixed-ratio combination analysis was performed, the relative contribution of each component cannot be determined from these data. Hence, the greater effect observed with the mixture cannot formally be ascribed to a synergistic interaction, because the study design does not allow the relative contribution of the individual components to be determined.

The concentrations of ITCs used in this study were selected with translational relevance in mind. Available pharmacokinetic data indicate that plasma concentrations in the upper micromolar range can be reached following oral intake of ITC-rich phytotherapeutic formulations (26), supporting the feasibility of the effective concentrations observed *in vitro*. In our *in vivo* experiments, the ITC mixture was administered via intraperitoneal (100 µL) or oral (200 µL) routes at 845 µM. The doses used here fall within, or below, the range used in prior animal studies examining the antiplatelet properties of single ITCs. For example: AITC administered at 3 mg/kg i.p. protected against pulmonary thromboembolism and prolonged bleeding time in mice (41), isopropyl ITC at 20–40 mg/kg orally reduced thrombosis (42), and SFN at 10–20 mg/kg inhibited occlusive thrombosis *in vivo* (40). Notably, the estimated ITC mass delivered by the ITC mixture (approximately 0.45 mg/kg per intraperitoneal and 0.89 mg/kg per oral administration; **Supplementary Table S2**) is lower than the doses reported for individual compounds in those studies. Because those experiments differ in model, route and schedule, and because plasma and tissue ITC concentrations were not measured here, these figures cannot be used to compare potency across studies. Pharmacokinetic and pharmacodynamic studies are required to establish the dose-exposure relationship for the mixture.

Mechanistically, these findings indicate that the ITC mixture inhibits platelet activation through a broad, multi-target mode of action, rather than by blocking a single receptor or enzymatic step. Unlike most conventional antiplatelet agents, the ITC mixture concurrently attenuates signaling outputs across multiple pathways essential for platelet activation. Kinome profiling identified reduced phosphorylation signatures within networks downstream of receptor tyrosine kinases, G protein-coupled receptors, and serine/threonine kinase cascades, including MAPK- and PKC-associated pathways. Many of these signaling pathways converge on intracellular Ca^2+^ mobilization and integrin αIIbβ_3_ activation. In line with this convergence, ITC-pretreated platelets exhibited markedly blunted Ca^2+^ responses following stimulation. As Ca^2+^ signaling is critical for calmodulin activation, granule fusion, cytoskeletal remodeling, and integrin αIIbβ_3_ activation (57), the attenuation of Ca^2+^ mobilization offers a mechanistically coherent explanation for the profound functional impairment observed in ITC-treated platelets.

In line with this interpretation, the ITC mixture inhibited ADP-induced aggregation. This agonist not only activates platelets through P2Y_12_-Gi-dependent signaling, which stabilizes aggregation and promotes dense-granule release, but also via P2Y_1_-Gq-mediated Ca^2+^ mobilization (58, 59). The suppression of aggregation downstream of both receptors suggests that the ITC mixture does not selectively target Ca^2+^-dependent signaling alone, nor does it inhibit all aspects of ADP-driven platelet activation. Rather, the attenuation of responses arising from both P2Y_1_- and P2Y_12_-dependent pathways suggests that the ITC mixture may affect shared downstream signaling nodes, including Ca^2+^ handling and Gi-linked effector pathways, such as AKT-dependent signaling. This mechanism contrasts with that of other phytochemicals, such as 1,8-cineole, which enhance endogenous inhibitory pathways by elevating cAMP (45, 60, 61). Our data are consistent with a model in which the ITC mixture may dampen multiple activation-promoting signaling axes, rather than selectively modulating an individual receptor or pathway.

Beyond signaling inhibition, the oxidative and metabolic effects of the ITC mixture on platelets represent a distinctive and likely critical component of their action. Platelet activation is normally accompanied by a rapid metabolic shift, characterized by increased mitochondrial respiration, ATP production, and tightly regulated ROS generation that reinforces signaling (22). ITC mixture-treated platelets failed to mount this metabolic response and instead displayed hallmarks of mitochondrial dysfunction, including membrane depolarization and impaired complex I- and complex II-dependent respiration. These findings suggest that the ITC mixture interferes directly with mitochondrial function, thereby limiting the energetic and redox capacity required for sustained platelet activation.

As highly electrophilic molecules that readily react with cysteine residues via their -N=C=S group (62–64), ITCs likely target cysteine-rich mitochondrial proteins. Several subunits of mitochondrial complex I contain redox-sensitive thiols, and modification of these sites is known to impair electron transport. In support of this concept, BITC has been shown to disrupt mitochondrial respiration in other cell types through modification of respiratory chain components (33, 65). Additionally, complex I-driven respiration is particularly important for granule secretion in platelets (21). This background provides a mechanistic explanation for how the ITC mixture may suppress degranulation when present before platelet activation, while aggregation, partially supported by glycolytic ATP (66), can still occur to a limited extent.

Consistent with this model, ITC mixture treatment increased protein S-glutathionylation, indicating a shift in redox balance toward oxidative modification of platelet proteins. Such modifications have been reported to alter the function of cytoskeletal and integrin-associated proteins and to dampen platelet responsiveness (67). Notably, although the ITC mixture imposed a basal oxidative burden, it simultaneously suppressed the activation-associated oxidant signal. The reduction in this signal was reproduced using CellROX™ Orange, whose signal is not dependent on mitochondrial accumulation (**Supplementary Figure S2**, **Supporting Information**). This dual effect suggests that the ITC mixture disrupts redox-sensitive signaling in a nuanced manner, imposing oxidative stress that limits platelet responsiveness while preventing the controlled ROS signals required for signal amplification. The net result is an energetically and redox-compromised platelet state that is less responsive to activating stimuli.

Our findings also offer important insight into the reversibility of different platelet activation events. Granule secretion of mediators and surface-expressed markers such as P-selectin, ADP, or CCL5 cannot be retracted, representing a rapid and essentially irreversible step in platelet activation. Addition of the ITC mixture even 25 s after TRAP stimulation did not reduce P-selectin expression or diminish levels of secreted mediators. In contrast, post-activation addition of the ITC mixture produced aggregometry changes consistent with destabilization of early, still-forming aggregates. Because aggregation depends on sustained αIIbβ3-fibrinogen interactions and actomyosin-dependent contractile tension (68), interrupting this signaling can destabilize aggregates. Indeed, ITC mixture addition after stimulation led to partial reductions in PAC-1 binding and fibrinogen association, consistent with integrin deactivation and weakening of aggregate-supporting forces. Accordingly, the aggregometry traces are consistent with partial destabilization of aggregates once they have begun to form, although the extent to which the fall in light transmission reflects true physical disassembly rather than loss of single platelets from aggregates remains to be confirmed by orthogonal methods. This would be unusual among antiplatelet agents, since most inhibitors prevent aggregation but do not destabilize aggregates once formed, and true thrombolysis remains the domain of plasminogen activators (17, 19, 20). Conversely, the ITC mixture does not reverse granule secretion once it has occurred, whereas the aggregometry traces are consistent with destabilization of dynamic integrin-fibrinogen interactions and actomyosin-generated contractile forces that maintain aggregation. Because light transmission aggregometry cannot distinguish physical disassembly from changes in platelet number, shape or light scattering, true aggregate disassembly remains to be confirmed using an orthogonal method.

Notably, this destabilization of platelet aggregates occurs without inducing additional platelet hyperactivation, further distinguishing the ITC mixture from established antiplatelet therapies. While some GPIIb/IIIa antagonists such as abciximab can prevent aggregation and, in some contexts, promote disaggregation, other agents in this class, such as tirofiban, have been reported to enhance degranulation or surface activation markers despite blocking fibrinogen binding (51). Whether the antithrombotic efficacy demonstrated here extends to arterial thrombotic settings therefore remains to be directly established. This is nonetheless a relevant consideration, as the ITC mixture’s mechanism, particularly suppression of integrin αIIbβ_3_ activation, Ca^2+^ mobilization, and aggregate stability, would be expected to be of particular consequence under high-shear arterial conditions where platelet activation dominates occlusive thrombus growth (69). Validation in an arterial thrombosis model thus represents an important direction for future investigation.

It is also informative to contrast the ITC mixture used here with SFN, one of the most extensively studied isothiocyanates. SFN has demonstrated antiplatelet and antithrombotic effects in several experimental models (40, 70), but these effects appear more agonist-selective, with stronger inhibition of collagen-induced aggregation than of thrombin- or ADP-mediated responses (40). By comparison, the ITC mixture examined in this study inhibited platelet activation across all agonists tested and produced aggregometry changes consistent with destabilization of early, still-forming aggregates, a feature not reported in prior studies of SFN. These differences underscore the functional diversity among ITCs and support the rationale for using defined mixtures to achieve broader and more robust antiplatelet effects.

Beyond the acute antithrombotic activity described here, ITCs are of interest for cardiovascular disease more broadly. Higher intake of cruciferous vegetables has been associated with lower cardiovascular risk, and experimental work attributes part of this protection to ITC-mediated Nrf2 activation in the vessel wall, with downstream suppression of endothelial adhesion-molecule expression, reduced vascular oxidative stress as well as attenuated inflammatory signaling (24, 37, 38). Because atherothrombosis arises from the interplay of platelet hyperreactivity and vascular inflammation, an agent capable of both restraining platelet activation and mitigating endothelial inflammation could theoretically target both pillars of the disease. Protective effects of individual ITCs have likewise been reported in experimental models of cardiac injury, and the oral activity seen in our animal model, together with the established clinical use of an ITC-containing preparation in another indication, makes further evaluation practical. A clinical cardiovascular role for ITCs nonetheless remains to be defined and will require dedicated dose-ranging, pharmacokinetic and controlled clinical studies.

Despite their promising properties, these findings raise several important mechanistic and translational questions regarding the ITC mixture. Most notably, the precise molecular targets of ITCs in platelets have yet to be fully defined. Due to their strong reactivity with protein thiols, ITCs may covalently modify cysteine residues on signaling enzymes such as PKC or PI3K, as well as on regulatory proteins involved in integrin activation, including talin or integrin β-subunits. This intrinsic chemical reactivity also raises concerns about target selectivity, since ITCs may induce widespread thiol modifications that contribute to generalized redox-dependent effects in addition to pathway-specific signaling perturbations. Identifying ITC-adducted or glutathionylated platelet proteins will be critical for delineating direct molecular targets and distinguishing specific signaling effects from more global redox-mediated mechanisms. Another important consideration is the durability of ITC mixture-mediated inhibition. ITCs are rapidly metabolized *in vivo* through glutathione conjugation. This raises the question of whether platelet function recovers fully after clearance, or if redox-dependent modifications persist sufficiently enough to confer sustained inhibition. Addressing these uncertainties will be essential for optimizing dosing strategies and for fully understanding the clinical significance of the ITC mixture.

Several limitations of the present study should be stated explicitly. (i), the mixture and its individual components were compared at a matched total concentration, not at equipotent doses. No fixed-ratio dilution series or combination-index analysis was performed. Our data therefore show that the mixture is more effective than its parts under these conditions, but they do not establish pharmacological synergy. (ii), the apparent destabilization of preformed aggregates was assessed using light transmission aggregometry. This technique cannot distinguish physical disassembly from single-platelet dropout or from changes in optical properties. Orthogonal confirmation is needed using imaging, flow-based sizing, clot retraction, or perfusion studies. (iii), the kinome data are predictive in nature. PamGene infers upstream kinase activity from peptide phosphorylation. Only the PI3K/AKT node was validated by immunoblot. The pathway-level directionality should therefore be treated as hypothesis-generating. (iv), the *in vivo* efficacy readout combines a quantitative tail-necrosis measurement with descriptive histopathology. No quantitative measure of thrombus burden was obtained, such as thrombus area, degree of vessel occlusion, or clot weight. In addition, the histological findings were not graded on a validated ordinal scale. (v), plasma and tissue ITC concentrations were not measured. The exposure achieved at the site of thrombus formation is therefore undefined. The apparent equivalence with heparin should be read as a comparison of endpoints within a single inflammation-driven venous model. It should not be interpreted as evidence of equivalent antithrombotic potency.

In summary, the data indicate that the ITC mixture, a defined combination of plant-derived isothiocyanates, constitutes a modulator of platelet function and thrombosis. By simultaneously suppressing upstream signaling pathways and promoting an oxidative-metabolic state that limits the capacity of platelets to sustain activation, the mixture operates through a mechanism distinct from those of current antiplatelet drugs. This distinctive profile suggests that the ITC mixture, or its optimized derivatives, may complement existing therapies by providing inhibition across multiple platelet activation pathways. While key mechanistic and translational questions remain to be addressed, these findings establish a strong foundation for further investigation of ITC mixture-based strategies and underscore their potential relevance in the modulation of platelet-driven thrombotic disease.

## Data Availability

The original contributions presented in this study are publicly available. The kinome profiling datasets (PamGene PamChip STK and PTK arrays) and associated metadata are available on Zenodo at https://zenodo.org/records/22713524. All other data supporting the conclusions of this article are included in the article and its **Supplementary Material**.

## Supplementary material

Supplementary Figure 1 Lactate Dehydrogenase (LDH) Release Assay. Plasma membrane integrity was evaluated by quantifying lactate dehydrogenase (LDH) release using the LDH-Glo™ Cytotoxicity Assay (J2380, Promega). Washed platelets (2 × 10^8^ platelets/mL) were incubated at 37 °C with varying concentrations of the ITC mixture (84.5, 253, 422, 845, or 2530 µM) or with a matched vehicle control in Tyrode’s buffer. Supernatants were harvested at 5, 15, and 30 minutes to correspond with critical experimental timepoints: the 5-minute pre-treatment period used in aggregation assays, the 15-minute (900-second) incubation for immunoblotting, and the 30-minute duration of the high-resolution respirometry protocol. Supernatants were cleared by centrifugation (2500 × g, 5 minutes, room temperature) and diluted in LDH storage buffer. Equal volumes of diluted supernatant and LDH Detection Reagent were combined, incubated for 30 minutes at room temperature protected from light, and luminescence was measured using Revvity Nivo multimode plate reader. Maximum LDH release (100% cytotoxicity) was determined by lysing vehicle-treated platelets with Triton X-100. Cytotoxicity for each sample was calculated as a percentage of this maximum after subtracting the background signal from the buffer control. The results were analyzed using a two-way repeated-measures ANOVA followed by Tukey’s multiple comparisons test at each individual timepoint. The assay was conducted in accordance with the manufacturer’s instructions. The ITC mixture does not compromise platelet plasma-membrane integrity at the working concentration. LDH release from washed human platelets exposed to the ITC mixture at 84.5, 253, 422, 845 or 2530 µM, or to the matched vehicle control, measured with the LDH-Glo™ Cytotoxicity Assay and expressed as percentage cytotoxicity relative to platelets lysed with 0.5% Triton X-100. Supernatants were sampled after 5, 15 and 30 minutes, spanning the 5-minute pre-treatment interval used in the aggregation experiments, the 15-minute (900-second) incubation used for immunoblotting, and the 30-minute duration of the high-resolution respirometry protocol. Data are mean ± SEM from at least 3 independent donors, as detailed in **Supplementary Table S1**. At the working concentration of 845 µM, LDH release did not differ significantly from vehicle control at any timepoint, and the same applied to 84.5, 253 and 422 µM at every timepoint. In contrast, 2530 µM, the concentration already shown to increase Annexin V binding (**Figure 1D**), produced a marked and time-dependent rise in LDH release at every timepoint. Statistical significance was determined by two-way repeated-measures ANOVA followed by Tukey’s multiple comparisons test within each timepoint ** p<0.005, **** p<0.0001.

Supplementary Figure 2 Intracellular ROS Detection with CellROX™ Orange. To circumvent the confounding effects of mitochondrial membrane potential (Δψm) on the retention of CellROX™ Green, intracellular oxidant levels were reassessed using CellROX™ Orange (C10443, Thermo Fisher). Unlike its green counterpart, this cell-permeant probe is retained in the cytosol upon oxidation and does not accumulate in the nucleus or mitochondria, rendering its fluorescence signal independent of Δψm. Ninety-six-well plates were coated with Cell-Tak™ (2 µg/cm²) according to the manufacturer’s instructions. Washed platelets (2 × 10^7^ platelets/100 µL) were then added to the coated wells and subjected to the same treatment conditions as in the CellROX™ Green experiments: vehicle control, ITC mixture (845 µM) alone, TRAP-6 (25 µM) alone, ITC mixture added 5 minutes prior to TRAP, or ITC mixture added 25 or 300 s post-TRAP, yielding total incubation times of 325 and 600 s, respectively. Following treatment, CellROX™ Orange was added to a final concentration of 1 µM, and platelets were incubated for 30 minutes at 37 °C in the dark. After incubation, wells were gently washed with PBS to remove unbound dye and non-adherent platelets. Fluorescence intensity was then measured using a Revvity Nivo multimode plate reader equipped with a 545/10 nm excitation filter and a 565/10 nm emission filter, according to the manufacturer’s specifications. All samples were assayed in 3 technical replicates, and the fluorescence values (relative fluorescence units, RFU) were background-subtracted and normalized to the paired vehicle control for each donor. CCCP was used as control to show that CellROX™ Orange is mitochondrial membrane potential independent, ROSUP (#C0177-2, Targetmol) was used as positive control. Data are presented as mean normalized fluorescence intensity ± SEM. Suppression of the activation-associated oxidant signal by the ITC mixture is independent of the mitochondrial membrane potential. Intracellular oxidant levels in washed human platelets measured by plate-reader fluorescence using CellROX™ Orange, a cytosolic probe that does not accumulate in mitochondria and whose retention is therefore not contingent on Δψm. Platelets were left untreated (vehicle control), exposed to the ITC mixture (845 µM) alone, stimulated with TRAP-6 (25 µM) alone, pre-treated with the ITC mixture for 5 minutes before TRAP, or stimulated with TRAP and given the ITC mixture 25 or 300 s later (total incubation 325 and 600 s, respectively). Data are background-subtracted relative fluorescence units (RFU) normalized to the paired vehicle control of the same donor and are shown as mean ± SEM from at least 3 independent donors, as detailed in **Supplementary Table S1**. TRAP alone increased the signal significantly over control. ITC pre-treatment and ITC addition 25 s after TRAP did not differ from control, whereas ITC addition at 300 s remained elevated. Statistical significance was determined by one-way ANOVA with Dunnett’s multiple comparisons test against the vehicle control. **** p<0.0001.

Supplementary Figure 3 Effects of IITC and SFN on human platelet aggregation. The effects of indole-3-isothiocyanate (IITC) and sulforaphane isothiocyanate (SFN) on human platelet aggregation were further evaluated. Platelets were pretreated with IITC or SFN for 5 minutes and then stimulated with TRAP (25 µM). Unlike the pronounced inhibitory effect observed with the ITC mixture, both IITC and SFN at 845 µM produced only minimal effects on platelet aggregation. Aggregation traces were largely preserved, with only minor changes in maximal aggregation and area under the curve (AUC) compared to control conditions. Due to their limited inhibitory activity, IITC and SFN were excluded from subsequent functional experiments and from the ITC mixture. These results suggest that IITC and SFN do not contribute to the anti-aggregatory activity observed with the defined ITC mixture. Limited effects of IITC and SFN on platelet aggregation. Representative light transmission aggregometry traces are shown for TRAP (25 µM)-stimulated human platelets following 5 minutes pretreatment with vehicle control, the ITC mixture, IITC, or SFN. AUC was quantified for each condition. In contrast to the ITC mixture, IITC and SFN exerted only minimal inhibitory effects on platelet aggregation. Data represent mean ± SEM from at least 3 independent biological replicates, as detailed in **Supplementary Table S1**. Statistical significance was determined by one-way ANOVA followed by Dunnett’s *post hoc* test. ****p < 0.0001.

Supplementary Figure 4 Time-dependent effects of ITC mixture treatment on agonist-induced platelet aggregation. Time-dependent effects of ITC mixture treatment on agonist-induced platelet aggregation. Human platelets were stimulated with distinct platelet agonists, and aggregation was assessed by light transmission aggregometry. Platelets were treated with the ITC mixture (845 µM), added either 25 s or 300 s after agonist stimulation. Activation was induced using **(A)** arachidonic acid (AA), **(B)** IV.3-mediated FcγRIIa crosslinking, **(C)** phorbol 12-myristate 13-acetate (PMA), **(D)** convulxin (CVX), or **(E)** U46619. Aggregation traces are expressed as percentage light transmission over time (mean ± SEM), and bar graphs depict aggregation quantified as area under the curve (AUC). Data represent mean ± SEM from at least 3 independent biological replicates, as detailed in **Supplementary Table S1**. Statistical significance was determined by one-way ANOVA followed by Dunnett’s *post hoc* test. ****p < 0.0001.

Supplementary Figure 5 Effects of ITC mixture treatment on agonist-induced platelet surface activation. Effects of ITC mixture treatment on agonist-induced platelet surface activation. Human platelets were stimulated with distinct platelet agonists, and surface activation was assessed by flow cytometric quantification of CD40L, CD63, and CD62P expression. Platelets were treated with the ITC mixture (845 µM), added either 25 s or 300 s after agonist stimulation. Activation was induced using **(A)** convulxin (CVX), **(B)** IV.3-mediated FcγRIIa crosslinking, **(C)** U46619, **(D)** phorbol 12-myristate 13-acetate (PMA), or **(E)** arachidonic acid (AA). Data represent mean ± SEM from at least 3 independent biological replicates, as detailed in **Supplementary Table S1**. Statistical significance was determined by one-way ANOVA followed by Dunnett’s *post hoc* test. *p < 0.05; **p < 0.005; ***p < 0.0005; ****p < 0.0001.

Supplementary Figure 6 Histopathological assessment of systemic tissue injury in carrageenan-induced thrombosis following ITC mixture or heparin treatment. Histopathological assessment of systemic tissue injury in carrageenan-induced thrombosis following ITC mixture or heparin treatment. Representative H&E-stained sections of heart, kidney, lung, liver, and tail collected 48 hours after carrageenan administration from mice in treatment groups T1-T11. Scale bars, 100 µm. **(A)** PBS-treated control mice (T1) display preserved tissue architecture across all examined organs. **(B)** Carrageenan-treated mice (T2) show widespread pathological changes, including myocardial and tail muscle necrosis, pulmonary vascular congestion with inflammatory infiltrates, and mild hepatocellular swelling. **(C–E)** Heparin-treated groups (T3-T5) exhibit partial protection from carrageenan-induced injury, with reduced cardiac and pulmonary thrombotic lesions, but residual changes such as focal hemorrhage, congestion, or mild hepatic and renal alterations, are seen in some animals. **(F–H)** ITC mixture-treated groups (T6-T8), administered either prophylactically or therapeutically, show substantial preservation of tissue architecture, marked reduction in cardiac and pulmonary thrombotic lesions, and attenuation of tail necrosis, with only mild focal changes observed in a subset of tissues. I-K) Oral ITC mixture treatment regimens (T9-T11) similarly maintain largely normal histology across organs, with minimal residual alterations, most commonly limited to mild pulmonary or renal changes. A summary of histopathological findings across all treatment groups is provided in **Table 2**.

Supplementary Table 1 Replicates, statistical tests and outlier exclusions per panel. Number of independent biological replicates (independent donors for the human platelet experiments, individual animals for the *in vivo* experiments), statistical test applied, and number of data points removed by the ROUT test (Q = 1%) for each panel. Replicate numbers and statistical tests are those stated in the corresponding figure legends; the ROUT test identified no outliers in any assay, so no data points were excluded.

Supplementary Table 2 Composition of the ITC mixture and delivered doses. Per-compound concentrations of the defined ITC mixture used in the cell-based experiments, and the corresponding amounts and doses delivered in the animal experiments. Doses are calculated for a body weight of 25 g. Additional concentrations used: Dose-response experiments (**Figures 1B–D**): total ITC mixture concentrations of 84.5, 253, 422, 845 and 2530 µM, each at the fixed 37.9: 50.0: 12.1 ratio. Single agents (**Figures 1G, H**): AITC, BITC or PEITC, each at 845 µM. Pairwise combinations (**Figures 1E, F**): AITC+BITC, AITC+PEITC and BITC+PEITC, each at a total concentration of 845 µM with the two components in the same relative proportion as in the complete mixture. Comparator isothiocyanates (**Supplementary Figure S3**, **Supporting information**): SFN and IITC, each at 845 µM. Vehicle control: dilution buffer with matched DMSO (< 0.2% v/v). Animal experiments: 845 µM (a 1:100 dilution of the 84.5 mM stock), 100 µL i.p. or 200 µL p.o.
