# Supplementary material for: A defined isothiocyanate mixture suppresses platelet signaling and metabolism to limit thrombosis in vivo
Source: Front Immunol. 2026 Sep 30;17:1944710. doi: 10.3389/fimmu.2026.1944710 (PMC13661427; doi:10.3389/fimmu.2026.1944710)
Supplement: Supplementary file 1 [file Supplementaryfile1.docx]

**Supplementary Figure 1.** Lactate Dehydrogenase (LDH) Release Assay

Plasma membrane integrity was evaluated by quantifying lactate dehydrogenase (LDH) release using the LDH-Glo™ Cytotoxicity Assay (J2380, Promega). Washed platelets (2 × 10⁸ platelets/mL) were incubated at 37°C with varying concentrations of the ITC mixture (84.5, 253, 422, 845, or 2530 µM) or with a matched vehicle control in Tyrode’s buffer. Supernatants were harvested at 5, 15, and 30 minutes to correspond with critical experimental timepoints: the 5-minute pre-treatment period used in aggregation assays, the 15-minute (900-second) incubation for immunoblotting, and the 30-minute duration of the high-resolution respirometry protocol. Supernatants were cleared by centrifugation (2500 × g, 5 minutes, room temperature) and diluted in LDH storage buffer. Equal volumes of diluted supernatant and LDH Detection Reagent were combined, incubated for 30 minutes at room temperature protected from light, and luminescence was measured using Revvity Nivo multimode plate reader. Maximum LDH release (100% cytotoxicity) was determined by lysing vehicle-treated platelets with Triton X-100. Cytotoxicity for each sample was calculated as a percentage of this maximum after subtracting the background signal from the buffer control. The results were analyzed using a two-way repeated-measures ANOVA followed by Tukey’s multiple comparisons test at each individual timepoint. The assay was conducted in accordance with the manufacturer's instructions.

**Figure S1. The ITC mixture does not compromise platelet plasma-membrane integrity at the working concentration.** LDH release from washed human platelets exposed to the ITC mixture at 84.5, 253, 422, 845 or 2530 µM, or to the matched vehicle control, measured with the LDH-Glo™ Cytotoxicity Assay and expressed as percentage cytotoxicity relative to platelets lysed with 0.5% Triton X-100. Supernatants were sampled after 5, 15 and 30 minutes, spanning the 5-minute pre-treatment interval used in the aggregation experiments, the 15-minute (900-second) incubation used for immunoblotting, and the 30-minute duration of the high-resolution respirometry protocol. Data are mean ± SEM from at least 3 independent donors, as detailed in Supplementary Table S1. At the working concentration of 845 µM, LDH release did not differ significantly from vehicle control at any timepoint, and the same applied to 84.5, 253 and 422 µM at every timepoint. In contrast, 2530 µM, the concentration already shown to increase Annexin V binding (Figure 1D), produced a marked and time-dependent rise in LDH release at every timepoint. Statistical significance was determined by two-way repeated-measures ANOVA followed by Tukey’s multiple comparisons test within each timepoint ** p<0.005, **** p<0.0001.

**Supplementary Figure 2.** Intracellular ROS Detection with CellROX™ Orange

To circumvent the confounding effects of mitochondrial membrane potential (Δψm) on the retention of CellROX™ Green, intracellular oxidant levels were reassessed using CellROX™ Orange (C10443, Thermo Fisher). Unlike its green counterpart, this cell-permeant probe is retained in the cytosol upon oxidation and does not accumulate in the nucleus or mitochondria, rendering its fluorescence signal independent of Δψm. Ninety-six-well plates were coated with Cell-Tak™ (2 µg/cm²) according to the manufacturer's instructions. Washed platelets (2 × 10⁷ platelets/100 µL) were then added to the coated wells and subjected to the same treatment conditions as in the CellROX™ Green experiments: vehicle control, ITC mixture (845 µM) alone, TRAP-6 (25 µM) alone, ITC mixture added 5 minutes prior to TRAP, or ITC mixture added 25 or 300 s post-TRAP, yielding total incubation times of 325 and 600 s, respectively. Following treatment, CellROX™ Orange was added to a final concentration of 1 µM, and platelets were incubated for 30 minutes at 37°C in the dark. After incubation, wells were gently washed with PBS to remove unbound dye and non-adherent platelets. Fluorescence intensity was then measured using a Revvity Nivo multimode plate reader equipped with a 545/10 nm excitation filter and a 565/10 nm emission filter, according to the manufacturer's specifications. All samples were assayed in 3 technical replicates, and the fluorescence values (relative fluorescence units, RFU) were background-subtracted and normalized to the paired vehicle control for each donor. CCCP was used as control to show that CellROX™ Orange is mitochondrial membrane potential independent, ROSUP (#C0177-2, Targetmol) was used as positive control. Data are presented as mean normalized fluorescence intensity ± SEM.

**Figure S2**. **Suppression of the activation-associated oxidant signal by the ITC mixture is independent of the mitochondrial membrane potential.** Intracellular oxidant levels in washed human platelets measured by plate-reader fluorescence using CellROX™ Orange, a cytosolic probe that does not accumulate in mitochondria and whose retention is therefore not contingent on Δψm. Platelets were left untreated (vehicle control), exposed to the ITC mixture (845 µM) alone, stimulated with TRAP-6 (25 µM) alone, pre-treated with the ITC mixture for 5 minutes before TRAP, or stimulated with TRAP and given the ITC mixture 25 or 300 s later (total incubation 325 and 600 s, respectively). Data are background-subtracted relative fluorescence units (RFU) normalized to the paired vehicle control of the same donor and are shown as mean ± SEM from at least 3 independent donors, as detailed in Supplementary Table S1. TRAP alone increased the signal significantly over control. ITC pre-treatment and ITC addition 25 s after TRAP did not differ from control, whereas ITC addition at 300 s remained elevated. Statistical significance was determined by one-way ANOVA with Dunnett’s multiple comparisons test against the vehicle control. **** p<0.0001

**Supplementary Figure 3.** Effects of IITC and SFN on human platelet aggregation

The effects of indole-3-isothiocyanate (IITC) and sulforaphane isothiocyanate (SFN) on human platelet aggregation were further evaluated. Platelets were pretreated with IITC or SFN for 5 minutes and then stimulated with TRAP (25 µM). Unlike the pronounced inhibitory effect observed with the ITC mixture, both IITC and SFN at 845 µM produced only minimal effects on platelet aggregation. Aggregation traces were largely preserved, with only minor changes in maximal aggregation and area under the curve (AUC) compared to control conditions. Due to their limited inhibitory activity, IITC and SFN were excluded from subsequent functional experiments and from the ITC mixture. These results suggest that IITC and SFN do not contribute to the anti-aggregatory activity observed with the defined ITC mixture.

**Figure S3. Limited effects of IITC and SFN on platelet aggregation.** Representative light transmission aggregometry traces are shown for TRAP (25 µM)-stimulated human platelets following 5 minutes pretreatment with vehicle control, the ITC mixture, IITC, or SFN. AUC was quantified for each condition. In contrast to the ITC mixture, IITC and SFN exerted only minimal inhibitory effects on platelet aggregation. Data represent mean ± SEM from at least 3 independent biological replicates, as detailed in Supplementary Table S1. Statistical significance was determined by one-way ANOVA followed by Dunnett’s post hoc test. ****p < 0.0001.

**Supplementary Figure 4. Time-dependent effects of ITC mixture treatment on agonist-induced platelet aggregation.**

**Figure S4. Time-dependent effects of ITC mixture treatment on agonist-induced platelet aggregation.** Human platelets were stimulated with distinct platelet agonists, and aggregation was assessed by light transmission aggregometry. Platelets were treated with the ITC mixture (845 µM), added either 25 s or 300 s after agonist stimulation. Activation was induced using A) arachidonic acid (AA), B) IV.3-mediated FcγRIIa crosslinking, C) phorbol 12-myristate 13-acetate (PMA), D) convulxin (CVX), or E) U46619. Aggregation traces are expressed as percentage light transmission over time (mean ± SEM), and bar graphs depict aggregation quantified as area under the curve (AUC). Data represent mean ± SEM from at least 3 independent biological replicates, as detailed in Supplementary Table S1. Statistical significance was determined by one-way ANOVA followed by Dunnett’s post hoc test. ****p < 0.0001.

**Supplementary Figure 5. Effects of ITC mixture treatment on agonist-induced platelet surface activation**.

**Figure S5. Effects of ITC mixture treatment on agonist-induced platelet surface activation**. Human platelets were stimulated with distinct platelet agonists, and surface activation was assessed by flow cytometric quantification of CD40L, CD63, and CD62P expression. Platelets were treated with the ITC mixture (845 µM), added either 25 s or 300 s after agonist stimulation. Activation was induced using A) convulxin (CVX), B) IV.3-mediated FcγRIIa crosslinking, C) U46619, D) phorbol 12-myristate 13-acetate (PMA), or E) arachidonic acid (AA). Data represent mean ± SEM from at least 3 independent biological replicates, as detailed in Supplementary Table S1. Statistical significance was determined by one-way ANOVA followed by Dunnett’s post hoc test. *p < 0.05; **p < 0.005; ***p < 0.0005; ****p < 0.0001.

**Supplementary Figure 6. Histopathological assessment of systemic tissue injury in carrageenan-induced thrombosis following ITC mixture or heparin treatment**

**Figure S6. Histopathological assessment of systemic tissue injury in carrageenan-induced thrombosis following ITC mixture or heparin treatment.** Representative H&E-stained sections of heart, kidney, lung, liver, and tail collected 48 hours after carrageenan administration from mice in treatment groups T1-T11. Scale bars, 100 µm. **A)** PBS-treated control mice (T1) display preserved tissue architecture across all examined organs. **B)** Carrageenan-treated mice (T2) show widespread pathological changes, including myocardial and tail muscle necrosis, pulmonary vascular congestion with inflammatory infiltrates, and mild hepatocellular swelling. **C-E)** Heparin-treated groups (T3-T5) exhibit partial protection from carrageenan-induced injury, with reduced cardiac and pulmonary thrombotic lesions, but residual changes such as focal hemorrhage, congestion, or mild hepatic and renal alterations, are seen in some animals. **F-H)** ITC mixture-treated groups (T6-T8), administered either prophylactically or therapeutically, show substantial preservation of tissue architecture, marked reduction in cardiac and pulmonary thrombotic lesions, and attenuation of tail necrosis, with only mild focal changes observed in a subset of tissues. **I-K)** Oral ITC mixture treatment regimens (T9-T11) similarly maintain largely normal histology across organs, with minimal residual alterations, most commonly limited to mild pulmonary or renal changes. A summary of histopathological findings across all treatment groups is provided in **Table 2**.

**Supplementary Table S1. Replicates, statistical tests and outlier exclusions per panel.** Number of independent biological replicates (independent donors for the human platelet experiments, individual animals for the *in vivo* experiments), statistical test applied, and number of data points removed by the ROUT test (Q = 1%) for each panel. Replicate numbers and statistical tests are those stated in the corresponding figure legends; the ROUT test identified no outliers in any assay, so no data points were excluded.

| Panel | Assay | n | Statistical test | Excluded by ROUT |
| --- | --- | --- | --- | --- |
| Figure 1B-C | LTA, ITC dose-response, TRAP | 4 | One-way ANOVA, Dunnett's post hoc test | 0 |
| Figure 1D | Annexin V binding (5 min) | 3 | One-way ANOVA, Dunnett's post hoc test | 0 |
| Figure 1E-F | LTA, pairwise ITC combinations | 5 | One-way ANOVA, Dunnett's post hoc test | 0 |
| Figure 1G-H | LTA, single ITCs | 4 | One-way ANOVA, Dunnett's post hoc test | 0 |
| Figure 1I-J | LTA, AA | 4 | One-way ANOVA, Dunnett's post hoc test | 0 |
| Figure 1I-J | LTA, U46619 | 4 | One-way ANOVA, Dunnett's post hoc test | 0 |
| Figure 1I-J | LTA, IV.3 | 4 | One-way ANOVA, Dunnett's post hoc test | 0 |
| Figure 1I-J | LTA, convulxin (CVX) | 3 | One-way ANOVA, Dunnett's post hoc test | 0 |
| Figure 1I-J | LTA, PMA | 3 | One-way ANOVA, Dunnett's post hoc test | 0 |
| Figure 1K-L | LTA, ADP in PRP | 3 | One-way ANOVA, Dunnett's post hoc test | 0 |
| Figure 2B | LTA, delayed ITC addition, TRAP | 7 | One-way ANOVA, Dunnett's post hoc test | 0 |
| Figure 2C | CD40L | 4 | One-way ANOVA, Dunnett's post hoc test | 0 |
| Figure 2C | CD63 | 4 | One-way ANOVA, Dunnett's post hoc test | 0 |
| Figure 2C | CD62P | 4 | One-way ANOVA, Dunnett's post hoc test | 0 |
| Figure 2D | CCL5 release, ELISA | 6 | One-way ANOVA, Dunnett's post hoc test | 0 |
| Figure 2E | Serotonin release | 6 | One-way ANOVA, Dunnett's post hoc test | 0 |
| Figure 2F | ATP release | 6 | One-way ANOVA, Dunnett's post hoc test | 0 |
| Figure 2G | PAC-1 binding | 4 | One-way ANOVA, Dunnett's post hoc test | 0 |
| Figure 2H | Fibrinogen binding | 4 | One-way ANOVA, Dunnett's post hoc test | 0 |
| Figure 3A-C | PamGene kinome profiling | 6 | One-way ANOVA, Dunnett's post hoc test | 0 |
| Figure 3D | pAKT (Ser473) immunoblot | 4 | One-way ANOVA, Dunnett's post hoc test | 0 |
| Figure 4A | Ca^2+^ mobilisation, Calbryte-520AM | 6 | One-way ANOVA, Dunnett's post hoc test | 0 |
| Figure 4B | CellROX™ Green | 6 | One-way ANOVA, Dunnett's post hoc test | 0 |
| Figure 4C | GSH / GSSG | 3 | One-way ANOVA, Dunnett's post hoc test | 0 |
| Figure 4D | Protein S-glutathionylation | 5 | One-way ANOVA, Dunnett's post hoc test | 0 |
| Figure 4E | TMRM, mitochondrial membrane potential | 7 | One-way ANOVA, Dunnett's post hoc test | 0 |
| Figure 4F | High-resolution respirometry | 6 | Unpaired Student's t-test | 0 |
| Figure 5B-D | TEM, mitochondrial morphometry | 4 donors, ≥50 mitochondria per donor | Unpaired Student's t-test | 0 |
| Figure 6C | Tail necrosis length | 5 mice | One-way ANOVA, Dunnett's post hoc test | 0 |
| Figure 6D | Blood loss (OD 490 nm) | 5 mice | One-way ANOVA, Dunnett's post hoc test | 0 |
| Figure 6E | Tail bleeding time | 5 mice | One-way ANOVA, Dunnett's post hoc test | 0 |
| Figure S1 | LDH release assay | 3 | Two-way ANOVA, Tukey’s post hoc test | 0 |
| Figure S2 | CellROX™ Orange assay | 3 | One-way ANOVA, Dunnett's post hoc test | 0 |
| Figure S3 | LTA, SFN and IITC | 3 | One-way ANOVA, Dunnett's post hoc test | 0 |
| Figure S4 | LTA, delayed ITC addition, other agonists | 3 | One-way ANOVA, Dunnett's post hoc test | 0 |
| Figure S5 | Surface activation markers, other agonists | 3 | One-way ANOVA, Dunnett's post hoc test | 0 |

**Supplementary Table S2. Composition of the ITC mixture and delivered doses.** Per-compound concentrations of the defined ITC mixture used in the cell-based experiments, and the corresponding amounts and doses delivered in the animal experiments. Doses are calculated for a body weight of 25 g.

| Compound | Proportion of mixture | Concentration at 845 µM total | Amount per mouse, i.p. (100 µL) | Dose, i.p. (mg/kg) | Dose, p.o. (mg/kg per administration) |
| --- | --- | --- | --- | --- | --- |
| AITC (allyl-ITC), M 99.15 g/mol | 37.9% | 320 µM | 3.17 µg | 0.13 | 0.25 |
| BITC (benzyl-ITC), M 149.21 g/mol | 50.0% | 422.5 µM | 6.30 µg | 0.25 | 0.50 |
| PEITC (phenethyl-ITC), M 163.24 g/mol | 12.1% | 102.5 µM | 1.67 µg | 0.07 | 0.13 |
| Total ITC mixture | 100% | 845 µM | 11.15 µg | 0.45 | 0.89 |

**Additional concentrations used:** Dose-response experiments (Figure 1B-D): total ITC mixture concentrations of 84.5, 253, 422, 845 and 2530 µM, each at the fixed 37.9 : 50.0 : 12.1 ratio. Single agents (Figure 1G-H): AITC, BITC or PEITC, each at 845 µM. Pairwise combinations (Figure 1E-F): AITC+BITC, AITC+PEITC and BITC+PEITC, each at a total concentration of 845 µM with the two components in the same relative proportion as in the complete mixture. Comparator isothiocyanates (**Figure S3**, **Supporting information**): SFN and IITC, each at 845 µM. Vehicle control: dilution buffer with matched DMSO (< 0.2% v/v). Animal experiments: 845 µM (a 1:100 dilution of the 84.5 mM stock), 100 µL i.p. or 200 µL p.o.
